# *Fgf9* regulates bone marrow mesenchymal stem cell fate and bone-fat balance in osteoporosis by PI3K/AKT/Hippo and MEK/ERK signaling

**DOI:** 10.7150/ijbs.94863

**Published:** 2024-06-17

**Authors:** Mingmei Chen, Hui Liang, Min Wu, Haoyang Ge, Yan Ma, Yan Shen, Shunyuan Lu, Chunling Shen, Hongxin Zhang, Zhugang Wang, Lingyun Tang

**Affiliations:** 1 State Key Laboratory of Medical Genomics, Research Center for Experimental Medicine, Rui-Jin Hospital affiliated to Shanghai Jiao Tong University School of Medicine, Shanghai, 200025, China.; 2 Shanghai Institute of Hematology, State Key Laboratory of Medical Genomics, National Research Center for Translational Medicine at Shanghai, Rui-Jin Hospital affiliated to Shanghai Jiao Tong University School of Medicine, Shanghai, 200025, China.; 3 Ruijin Hospital Lu Wan Branch, Shanghai Jiaotong University School of Medicine, Shanghai, 200025, China.

**Keywords:** Adipogenesis, Bone-fate balance, Bone marrow adipose tissue, Mesenchymal stem cells, Osteogenesis

## Abstract

Bone-fat balance is crucial to maintain bone homeostasis. As common progenitor cells of osteoblasts and adipocytes, bone marrow mesenchymal stem cells (BMSCs) are delicately balanced for their differentiation commitment. However, the exact mechanisms governing BMSC cell fate are unclear. In this study, we discovered that fibroblast growth factor 9 (*Fgf9*), a cytokine expressed in the bone marrow niche, controlled bone-fat balance by influencing the cell fate of BMSCs. Histomorphology and cytodifferentiation analysis showed that *Fgf9* loss-of-function mutation (S99N) notably inhibited bone marrow adipose tissue (BMAT) formation and alleviated ovariectomy-induced bone loss and BMAT accumulation in adult mice. Furthermore, *in vitro* and *in vivo* investigations demonstrated that *Fgf9* altered the differentiation potential of BMSCs, shifting from osteogenesis to adipogenesis at the early stages of cell commitment. Transcriptomic and gene expression analyses demonstrated that FGF9 upregulated the expression of adipogenic genes while downregulating osteogenic gene expression at both mRNA and protein levels. Mechanistic studies revealed that FGF9, through FGFR1, promoted adipogenic gene expression via PI3K/AKT/Hippo pathways and inhibited osteogenic gene expression via MAPK/ERK pathway. This study underscores the crucial role of *Fgf9* as a cytokine regulating the bone-fat balance in adult bone, suggesting that *FGF9* is a potentially therapeutic target in the treatment of osteoporosis.

## Introduction

Mesenchymal stem cells derived from bone marrow can differentiate into osteoblasts and adipocytes in adult bones 1. The commitment to these lineages is inversely correlated, as the adipogenic differentiation of BMSCs requires a coordinated inhibition of osteogenic differentiation 2. The balance between osteogenic and adipogenic lineage commitment is critical for preserving bone homeostasis 3-5. Numerous investigations involving mouse models and humans have further corroborated the link between elevated marrow fat content, diminished bone density, and increased susceptibility to fractures, particularly in osteoporosis and skeletal aging 6-10. Therefore, regulating BMSCs fate determination and the delicate balance between bone and fat have garnered increasing attention in recent years 11-16. Nevertheless, mechanisms that determine the fate of BMSCs remain to be thoroughly elucidated. Over the past decade, several transcriptional factors associated with BMSCs fate commitment have been identified. Notably, *Runx2* and *Osterix* function as master regulators guiding differentiation toward the osteogenic lineage, while *Pparg* and *Cebpa/b/d* are considered essential for adipogenesis 4, 17. Considering the fate determination of BMSCs is strictly orchestrated by molecular signals emanating from the bone marrow microenvironment, it becomes apparent that, despite the identification of master regulators for osteoblastic and adipogenic lineages, the understanding of the associated factors during BMAT accumulation and osteoporotic bone loss within the microenvironment remains limited.

The Fibroblast Growth Factor (FGF) family is an important class of cytokines that plays a crucial role in maintaining bone homeostasis 18-21. The FGF family contains 22 genes encoding structure-related proteins, including canonical, hormone-like, and intracellular subfamilies 22. Secreted FGFs mediate their biological responses by selectively binding to and activating FGF receptors (FGFRs) 23, 24. Among them, there are three canonical FGFs (*Fgf7*, *Fgf9,* and *Fgf18*) and a hormone-like FGF (*Fgf23*) expressed in the adult bone marrow niche 25, 26. As one of the most studied members of the FGF family, *Fgf9* is confirmed with significance in multiple organ development, including the lung, testis, kidney, ear, pancreas, and skeletal system 27-33. However, its role in maintaining adult bone homeostasis is not fully understood. Due to the redundancy and compensatory effects among the 22 members of the FGF family, gene knockout can sometimes not fully reveal a specific gene's biological functions. Sometimes, a double-knockout mouse model was needed to observe the phenotype 33, 34. Previously, we have reported a missense mutation (Ser99Asp, S99N) in exon 2 of the *FGF9* gene leading to multiple synostoses syndrome 3 (SYNS3) 35, 36. This mutation exhibits both loss-of-function and dominant negative effects. *Fgf9^S99N^* mutation impaired the affinity between FGF9 and FGFRs and interfered with the affinity between wild-type FGF9 and other FGFs with heparan sulfate proteoglycan (HSPG), leading to weakened FGF signal transduction during joint development and resulting in the failure of synovial joint formation in *Fgf9^S99N^* knock-in mouse models 36, 37. Further investigation revealed that *Fgf9* negatively regulates bone mass via inhibiting osteogenesis and mineralization and promoting osteoclastogenesis 38. Interestingly, we observed that under osteogenic-induction conditions, recombinant FGF9 protein not only inhibited the differentiation of BMSCs into osteoblasts but also compelled their differentiation into adipocytes. Some *in vitro* studies have revealed that *Fgf9* inhibits the differentiation and mineralization of mesenchymal stem cells and osteoprogenitor cells 39-41. Moreover, recent studies reported that *Fgf9* also plays a regulatory role in brown adipogenesis 42, 43 and lipid metabolism 44, 45. This led us to hypothesize that *Fgf9* might play a crucial role in adult bone homeostasis by regulating the lineage commitment of BMSCs, and *Fgf9^S99N^* knock-in mouse models serve as a valuable model for studying the regulation of bone homeostasis by FGF9.

In this study, we found that *Fgf9* was expressed in the bone marrow niche and upregulated with aging and ovariectomy (OVX). As a previously proven loss-of-function mutation of *Fgf9*, we employed *Fgf9^S99N^* mutant mice, OVX-induced osteoporosis model, and cytodifferentiation assays to investigate the role of *Fgf9* in regulating bone and fat balance in adult bone. *Fgf9^S99N^* mutation significantly inhibited the formation of BMAT in adult mice and alleviated the OVX-induced bone loss and BMAT accumulation. *In vivo* and *in vitro* analysis showed that *Fgf9* significantly promoted adipocyte formation and inhibited osteogenesis of BMSCs at the early stages of differentiation. Mechanistic investigations revealed that *Fgf9* modified the expression of osteogenic and adipogenic genes via the MEK/ERK pathway and the PI3K/AKT/Hippo pathway. This study provides the first evidence that *Fgf9* plays a critical role in the fate determination of BMSCs by facilitating adipogenesis and inhibiting osteogenesis, suggesting that *FGF9* is a potential therapeutic target in the treatment of osteoporosis.

## Materials and methods

### Mice

Mice with the *Fgf9^S99N^* mutation were generated as previously described and bred into the C57BL/6 background 38. The animals were housed with free access to water and diet in specific pathogen-free (SPF) conditions (12 hours light-dark cycle, 21-23 ℃ and 60% relative humidity) at Ruijin Hospital, Shanghai Jiao Tong University (SJTU) School of Medicine, Shanghai, China. The control and mutant mice are same-sex siblings from the same littermates. BALB/c Nude mice (Cat. NO. SM-014) were purchased from Shanghai Model Organisms Center, Inc. All animal procedures were reviewed and approved by the Institutional Animal Care and Use Committee of Shanghai Jiao Tong University (approval number: SYXK 2023-0007).

### Ovariectomy

8-week-old female wild-type (wt) and heterozygotes (het) mice were anesthetized by intraperitoneal injection of pentobarbital sodium according to body weight. Mice in the OVX group underwent bilateral ovariectomy and tubal ligation under anesthesia. The sham group underwent a laparotomy with the removal of an equivalent amount of abdominal fat tissue. After two months of normal water and diet, the mice were euthanized. Blood samples were collected for serum separation to analyze PINP and CTX-1 levels. The left femurs and tibiae were harvested and fixed in 4% paraformaldehyde (PFA) at 4 °C for 48 hours. The right femurs and tibiae were collected for RNA extraction.

### Micro-CT

The micro-CT analysis followed the recommendations of the American Society for Bone and Mineral Research (ASBMR) 46. The femurs were scanned in PBS on a Skyscan 1275 (Bruker, Belgium) at an 8 μm resolution with 50 kV source voltage, 60 μA source current, 110 ms exposure, and 0.2º rotation step. Three-dimensional reconstruction of femurs were generated to analyze the trabecular and cortical bone microarchitecture. The region of interest (ROI) of trabecular bone was defined as a distance of 1.6 mm from the end of the growth plate towards the diaphysis. The cortical ROI was defined as a distance of 400 μm from the mid-diaphysis to the distal diaphysis. Trabecular bone parameters including Bone mineral density (BMD, mg/cc), Bone volume (BV, mm^3^), Percent bone volume (BV/TV, %), Bone surface (BS, mm^2^), Bone surface density (BS/TV, 1/mm), Trabecular number (Tb. N, 1/mm), Trabecular separation (Tb. Sp, mm) and Connectivity (Conn) were measured. Cortical bone parameters were measured, including Object volume (Obj. V, mm^3^) and Object surface (Obj. S, mm^2^).

### Bone histomorphometry and immunofluorescence staining

Femurs were fixed in 4% PFA at 4°C for 48 hours and then decalcified in 12.5% EDTA2Na (PH 7.4) solution at 4 °C for 2 weeks, with the solution changed every 3 days. Samples were embedded in paraffin, and 6-μm thick sections underwent histomorphometry analyses. H&E staining was performed with a Hematoxylin-Eosin staining kit (E607318, Sangon, China). TRAP staining was performed with the Tartrate-resistant acid phosphatase stain kit (S0102, Bioss, China). For immunofluorescence staining (IF), sections were deparaffinized and rehydrated, and underwent antigen retrieval. BMSCs or fibroblast-like cells were cultured on the slides and then fixed with 4% PFA. Sections and cells were blocked with 1% BSA in PBST (PBS with 0.5% Triton-100) at room temperature for 1 hour, followed by incubation at 4°C overnight with primary antibodies (see Table S3 for details). The next day, sections and cells were incubated with secondary antibodies at room temperature for 2 hours. After washing, these were covered with Mounting Medium with DAPI (ab104139, Abcam, England) for microscopic detection. All images were captured with a multifunctional imaging detector BioTek Cytation5 (Agilent, USA).

### BMSCs primary culture

The BMSCs were isolated from compact bone, following the experimental protocol described by Brenton J. Short et al 47. Mice were euthanized and soaked in 75% ethanol for 3 minutes. Under sterile conditions, the femurs and tibiae were separated, and the attached tissues were carefully removed with gauze. Cut both ends of the bone and flush the marrow cavity with a 23-gauge needle and 10 ml PBSFE (PBS with 2% FBS and 1 mM EDTA). Bone marrow-free bones were snipped and then cut into 1-2 mm bone fragments with a scalpel. Transferred the bone fragments to a 50 ml polypropylene tube with 2 ml/per mouse collagenase solution constituted with α-MEM medium, 10% FBS, 0.3% Collagenase I (C0130, Sigma-Aldrich, America), and 0.1% DNase I (10104159001, Roche, Germany) and incubated at 37 °C for 45 minutes with 200 rpm rotation. The supernatant was filtered by a 70 μm cell strainer and centrifuged at room temperature at 300g for 10 minutes. Carefully removed the supernatant and resuspended the cell pellet with 1ml culture medium (05513, STEMCELL, Canada). Expected cell recovery is 1.5-3.5×10^6^ cells per mouse. Count nucleated cells using trypan blue and seed the cells at 3-6×10^4^ cells/cm^2^ with culture medium. BMSCs were cultured at 37°C in a 5% CO_2_ incubator, and half of the medium was changed every two days. After 6-10 days, the clones were formed and expanded to sub-confluent (60-80%). BMSCs were generated at a split ratio of 1:3 and cultured for no more than three passages. The BMSCs were characterized using flow cytometry to detect cell surface markers (negative markers: CD31, CD34, CD45, and Ter-119; positive markers: CD29, CD44, Sca-1, CD140a).

### Bone marrow fibroblast-like cell primary culture

Euthanized mice were submerged in 75% ethanol for 3 minutes for disinfection. Under sterile conditions, the femurs and tibiae were dissected, and the muscle and connective tissue were gently removed using gauze. The bone ends were cut, and the bone marrow cavity was flushed with 10 ml PBSFE using a 23-gauge needle. The bone marrow was collected by centrifugation at 300g for 10 minutes. Next, red blood cells were lysed with red blood cell lysis buffer for 5 minutes at room temperature, followed by adding PBSFE at a 5 times volume. The cell suspension was filtered using a 70 μm cell strainer, followed by another centrifugation step at room temperature and 300g for 10 minutes. The cells were then resuspended and seeded into a 10 cm dish per mouse with culture medium. The cells were cultured at 37°C in a 5% CO2 incubator for 48 hours. Unattached cells were removed by medium change, and after that, half of the medium was replaced every two days. After 6-10 days, the fibroblast-like cells should reach a sub-confluent state of 60-80% confluency.

### *In vitro* cell differentiation

For cell differentiation, BMSCs were seeded at a density of 1-2×10^4^ cells/drop (15-20 μl/drop) on the center of the well in the 48-well plate and cultured at 37°C in a 5% CO_2_ incubator for two hours. After the BMSCs were attached, the culture medium was added and cultured for 24 hours. The culture medium was changed to osteogenic induction medium (OI) (α-MEM supplemented with 10% FBS, 10^-8^ M dexamethasone, 10 mM beta-glycerol phosphate and 50 μg/ml ascorbate-2-phosphate) or adipogenic induction medium (AI) (α-MEM supplemented with 10% FBS, 10^-6^ M dexamethasone, 1 μM Rosiglitazone, 0.5 mM 3-Isobutyl-1-methylxanthine and 2.5 μg/ml Insulin) with different concentrations of recombinant mouse FGF9 Protein (7399-F9, R&D Systems, USA). The medium was changed every two days until the desired differentiated status.

### ALP, Von Kossa and Oil red-O staining

BMSCs were fixed with 4% PFA and processed with ALP, Von Kossa, and Oil Red O staining. ALP staining was performed according to the NBT/BCIP kit instruction (11697471001, Roche, Germany). For Von Kossa staining, cells were incubated with 5% silver nitrate solution for 30 minutes and washed three times with ddH_2_O for 5 minutes each time. The mineralized nodules were developed with 1% pyrogallol for 2 minutes, washed with ddH_2_O, and air-dried. Oli Red-O staining for adipogenesis was performed using an Oli Red-O solution according to the manufacturer's instructions (O0625, Sigma-Aldrich, America). Images were captured under a multifunctional imaging detector BioTek Cytation5 (Agilent, USA) and analyzed with Image J software.

### *Fgf9* overexpression in BMSCs

The primary cultured BMSCs were isolated from 1-month-old mice as described above. Upon reaching 40% confluency, BMSCs were transfected with *OE-Fgf9* and *OE-control* lentivirus (LV5 EF-1a/GFP&Puro, 1×10^9^ TU/ml, 1 μl/cm^2^) and polybrene (5 μg/ml) for 48h. The non-transfected cells were eliminated by puromycin treatment. qRT-PCR verified overexpression of *Fgf9* in BMSCs. The transfected cells were used before passage 3.

### *In vivo* transplantation

The 4-week-old nude mice underwent subcutaneous injection of BMSCs. 5×10^5^ *OE-ctrl* BMSCs were injected into the scapular region behind the left axilla, and 5×10^5^ *OE-Fgf9* BMSCs were injected into the right scapular region of the same nude mouse. Mice were euthanized 5 weeks later. The skin and subcutaneous tissue were isolated and fixed in 4% paraformaldehyde at 4°C for 48 hours. The tissues were decalcified and subjected to H&E staining and immunofluorescent staining.

### qRT-PCR

Total RNA was extracted from mouse bones or cells according to the TriPure isolation reagent protocol (11667165001, Roche, Germany). cDNA was synthesized using PrimeScript™RT reagent Kit according to the manufacturer's instruction (RR037A, Takara, Japan). Gene expression levels were detected using QuantStudio5 (Thermo Fisher Scientific, USA) with 2×SG Fast qPCR Master Mix (B639271, Sangon, China). The primers used are presented in Table S2. β-actin was used as a normalization control.

### Western blot

Cells or femurs were lysed in RIPA Lysis Buffer (C500005, Sangon, China) containing cOmplete™ Mini protease inhibitor (04693124001, Roche, Germany) and PhosSTOP™ phosphatase inhibitor (04906837001, Roche, Germany). The samples were separated by SDS-PAGE gel and transferred to nitrocellulose blotting membranes. After blotting with 5% skim milk for 1 hour, the membranes were incubated with primary antibodies at 4°C overnight and fluorescent secondary antibodies at room temperature for 2 hours (Table S3). The Odyssey near-infrared fluorescence imaging system (LI-COR, Lincoln, NE, USA) was used for imaging. GAPDH was used as the internal control.

### ELISA

Serum was obtained from OVX and sham mice. The serum level of PINP was analyzed using the Mouse PINP (Procollagen1 N-Terminal Propeptide) ELISA Kit (D721053-0048, Sangon, China) according to the manufacturer's protocol. The serum level of CTX-1 was analyzed using the Mouse CTX-1 (Cross Linked C-telopeptide of Type1 Collagen) ELISA Kit (D721204-0048, Sangon, China) according to the manufacturer's protocol. ELISA was performed using the automatic microplate reader BioTek synergy H1 (Agilent, USA).

### Single-cell bioinformatics analysis

Single Cell PORTAL was used to analyze the bone marrow niche (https://singlecell.broadinstitute.org/single_cell/study/SCP1248/resolving-the-bone-marrow-niche-heterogeneity; https://singlecell.broadinstitute.org/single_cell/study/SCP361/mouse-bone-marrow-stroma-in-homeostasis) 25, 26. The t-Distributed stochastic neighbor embedding (t-SNE) algorithm was used for cell grouping. The expression of *Fgf9* and *Fgfrs* in bone marrow stroma clusters were analyzed and shown in the Dot plot.

### RNA-seq and transcriptome analysis

Eighteen samples derived from BMSCs were harvested for RNA-seq. Total RNA was extracted and then assessed by Agilent 2100 Bioanalyzer (Agilent Technologies, Germany). The cDNA libraries were constructed and arrayed for high-throughput sequencing with the Illumina Hiseq™ 3000 platform. Cutadapt V1.9.1 processed raw reads to be high-quality, clean data. The clean data were mapped to the reference genome via Hisat2 V2.2.1. Differential expression analysis was performed by the DESeq2 Bioconductor package (V1.6.3). The differentially expressed genes (DEGs) were defined as Log2 fold change absolute value≥1 and adjusted P-value≤0.05. Gene Ontology (GO) enrichment and Kyoto Encyclopedia of Genes and Genomes (KEGG) analysis were performed using clusterProfiler V3.0.5. In addition, Heatmaps, GO terms, and KEGG pathways were graphically drawn, and figures were displayed through the online bioinformatics analysis and visualization cloud platform (bioinformatics, http://www.bioinformatics.com.cn/). The RNA-seq datasets have been submitted to the NCBI database under the accession number GSE252394.

### Statistical analysis

In statistical analysis, each group of samples has at least three biological replicates. Data are expressed as box-and-whisker plots (with median and interquartile ranges) from max to min, with all data points shown unless otherwise noted. Unpaired, two-tailed Student's t-tests were used to determine significance, using p < 0.05 as the cutoff. All data were drawn using GraphPad Prism 7.0 software.

## Results

### *Fgf9* is expressed in the bone marrow niche and upregulated with aging and OVX

**Figure 1:**
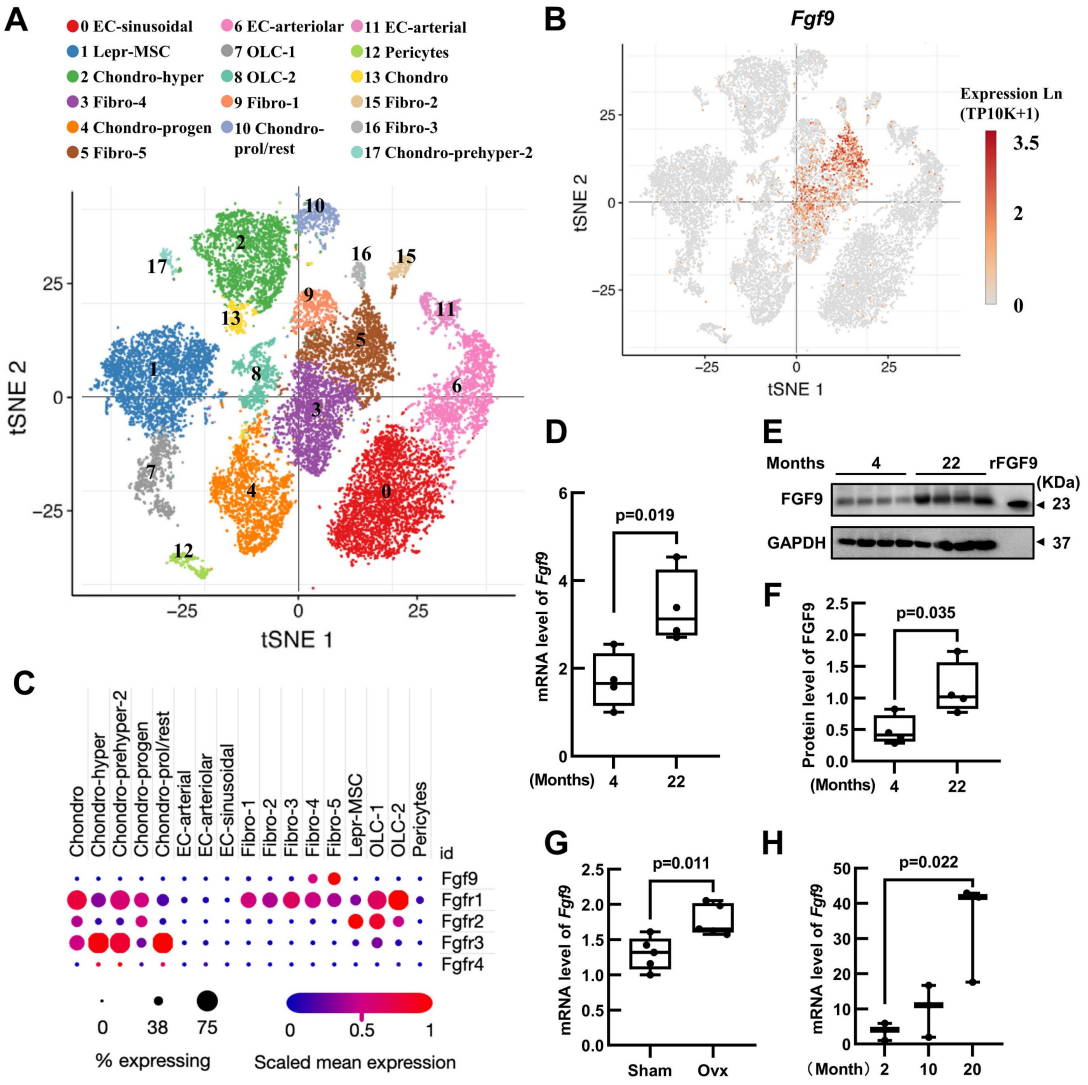
***Fgf9* is expressed in the bone marrow niche and upregulated with aging and OVX. (A)** UMAP visualization of seventeen bone marrow stroma cell clusters. **(B)** Distribution of *Fgf9* in bone marrow stroma cell clusters. **(C)** Expression of *Fgf9* and *Fgfrs* in seventeen clusters of bone marrow stroma cells. The size of dots represents the percentage of expression; red and blue represent the level of scaled mean expression. **(D)** Relative mRNA levels of *Fgf9* in tibiae of young (4-month-old) and aged (22-month-old) mice, n = 4 in each group. **(E, F)** Protein levels of FGF9 in femurs of young (4-month-old) and aged (22-month-old) mice were detected by immunoblotting (E) and were quantitatively analyzed (F), n = 4 in each group. Recombinant FGF9 protein (rFGF9) as the positive control. **(G)** Relative mRNA levels of *Fgf9* in tibiae of Sham-wt and OVX-wt mice (4-month-old), n = 5 in each group. **(H)** Relative mRNA levels of *Fgf9* in fibroblast-like cells of young (2-month-old), middle (10-month-old), and aged (20-month-old) mice, n = 3 in each group. Data are analyzed by Student's t-test and shown as boxplots (median ± interquartile range).

In order to elucidate the role of *Fgf9* in maintaining adult bone homeostasis, we used Single Cell PORTAL (https://singlecell.broadinstitute.org/single_cell) to search through two projects: "Mouse Bone Marrow Stroma in Homeostasis" (20581 cells, 27998 genes) 25 and " Resolving the Bone Marrow Niche Heterogeneity " (32743 cells, 17173 genes) 26. We analyzed the expression patterns of *Fgf9* and four receptors (*Fgfr1-4*) in the main cell populations within the bone marrow microenvironment. The results revealed that *Fgf9* is predominantly expressed in the fibroblasts subpopulation, *Fgfr1* is mainly expressed in fibroblasts, chondrocytes, and mesenchymal stem cells, *Fgfr2* shows primary expression in mesenchymal stem cells and Schwann cells, *Fgfr3* is primarily expressed in chondrocytes, and *Fgfr4* is expressed in myofibroblasts (Figure 1A-C; Figure S1A-C). To further assess the role of *Fgf9* in bone homeostasis, we examine the expression levels of the *Fgf9* gene in the skeletons of young (4 months) and elderly mice (22 months), as well as in OVX-induced and sham-operated control mice. The results showed a significant increase in both *Fgf9* mRNA and protein levels in the bone of elderly mice (Figure 1D-F), as well as a similar elevation in *Fgf9* mRNA levels in OVX-induced mice (Figure 1G). Additionally, we cultured bone marrow fibroblast-like cells from C57BL/6 mice of different ages (2, 10, 20 months). Immunofluorescence staining using S100A4, one of the markers for fibroblasts, revealed approximately 47% of cells were fibroblasts (Figure S1D and E). qRT-PCR analysis demonstrated a significant increase in the mRNA levels of *Fgf9* in the fibroblast-like cells with age (Figure 1H). These findings suggest that *Fgf9* is a growth factor specifically expressed in the adult bone marrow microenvironment, potentially playing a crucial role in maintaining bone homeostasis.

### The *Fgf9^S99N^* mutation alleviates the OVX-induced bone loss and BMAT accumulation

**Figure 2:**
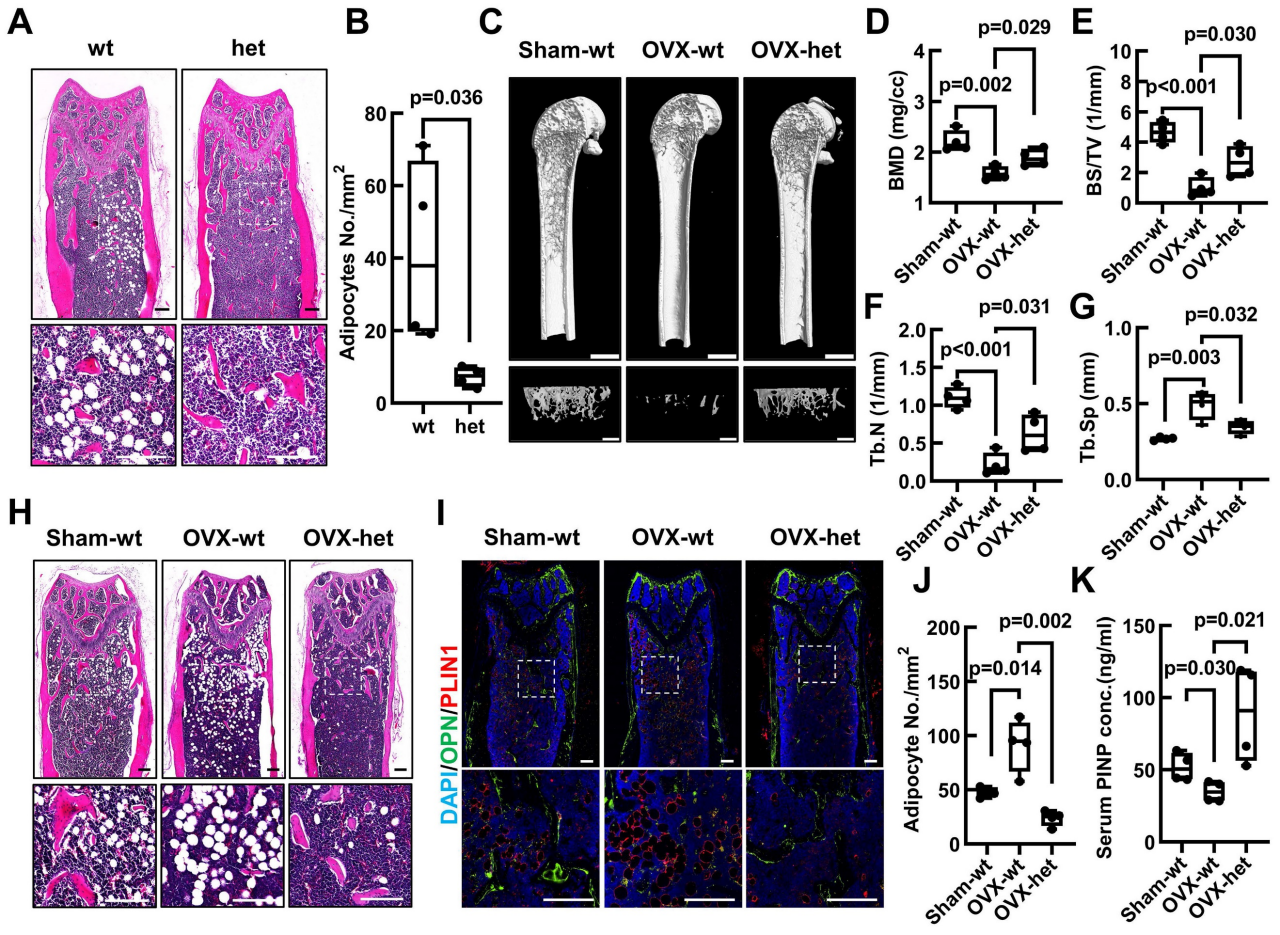
***Fgf9^S99N^* mutation mitigates bone-fat imbalance in OVX-induced osteoporosis. (A)** H&E staining of longitudinal section of femurs from 4-month-old male wild-type (wt) and heterozygous (het) mice.** (B)** Statistical analysis of femur adipocyte number in 4-month-old wt and het mice.** (C)** 8-week-old female wt and het mice underwent sham surgery and ovariectomy, and their femurs were assessed using micro-CT scanning after 8 weeks. The representative figures illustrate the 3D reconstructed structures, the sagittal section of the femur (scale bars = 2 mm) and the trabecular bone of the metaphyseal region (scale bars = 1 mm).** (D-G)** Bone mineral density (BMD), Bone surface density (BS/TV), Trabecular number (Tb.N), and Trabecular separation (Tb.Sp) of trabecular bone of Sham-wt, OVX-wt, and OVX-het mice were determined by micro-CT analysis.** (H)** Representative images of H&E staining in left femurs of Sham-wt, OVX-wt, and OVX-het mice.** (I)** Immunofluorescence staining of Osteopontin and Perilipin A in femurs of Sham-wt, OVX-wt, and OVX-het mice.** (J)** Statistical analysis of adipocyte number in femurs of Sham-wt, OVX-wt, and OVX-het mice.** (K)** Quantification analysis of serum PINP level in Sham-wt, OVX-wt, and OVX-het mice by ELISA. Data are analyzed by Student's t-test and shown as boxplots (median ± interquartile range), n = 4 mice in each group. In (A), (H), and (I), the dashed box indicates the area of local magnification (below), and the scale bars represent 200 μm.

To investigate the potential role of *Fgf9* in maintaining the balance between bone and fat tissue in bone, we examined the bone and BMAT formation in adult mice harboring *Fgf9^S99N^* mutation. Histological analysis employing Hematoxylin and Eosin (H&E) staining, along with statistical analysis, unequivocally demonstrated a significant reduction in bone marrow adipocytes within the femurs and tibiae of 4-month-old male *Fgf9^wt/mut^* mice in comparison to their wt male littermates (Figure 2A and B; Figure S2A-E). Moreover, the mRNA expression levels of adipocyte markers (*Adipoq* and *Leptin*) and osteogenic markers (*Alpl* and *Col1a1*) were detected by qRT-PCR in femurs of neonatal *Fgf9^wt/wt^*, *Fgf9^wt/mut^*, and *Fgf9^mut/mut^* mice. The results indicated a decrease in adipocyte marker expression alongside an increase in osteogenic marker expression (Figure S3I-L).

To further evaluate the potential contribution of *Fgf9* towards the bone-fat balance, the *Fgf9^wt/mut^* mice and wt counterparts underwent the OVX surgery, which serves as a classical model to simulate postmenopausal osteoporosis characterized by BMAT accumulation and bone loss 48. Micro-computed tomography (micro-CT) analysis of the femurs revealed a notable reduction in trabecular and cortical bone in 4-month-old wt mice subjected to OVX (Figure 2C; Figure S3E). Intriguingly, *Fgf9^wt/mu^*^t^ mice exhibited significant resistance to the OVX-induced bone loss (Figure 2C; Figure S3E). Further quantitative evaluation of bone structural parameters, encompassing femoral trabecular parameters (BMD, BV, BS, BV/TV, Tb. N, Tb. Sp, Conn.,) (Figure 2D-G; Figure S3A-D) and cortical bone parameters (Obj. V and Obj. S) (Figure S3F-G), further confirmed these observations. Correspondingly, the serum concentrations of PINP indicated a significant increase in osteoblastic activity in OVX-het mice compared to OVX-wt mice (Figure 2K). Alternatively, the histological analysis uncovered that the *Fgf9^S99N^* mutation mitigated the BMAT accumulation after OVX surgery. H&E staining and quantitative analysis revealed that OVX-wt mice showed obvious bone marrow adiposity characterized by fat vacuoles compared with Sham-wt mice, and the heterozygous mice significantly ameliorated BMAT accumulation with OVX-induction (Figure 2H and J; Figure S3H). Immunofluorescence staining of lipid droplet marker PerilipinA/PLIN1 and bone marker Osteopontin/OPN demonstrated diminished BMAT and enhanced bone formation in femurs of OVX-het mice compared to OVX-wt mice (Figure 2I). Given the importance of bone resorption in maintaining bone homeostasis, it is crucial to investigate the influence of FGF9 on osteoclast activity. We evaluated serum CTX-I levels in Sham and OVX mice and found no significant difference between the OVX-wt and OVX-het groups (Figure S3M). Furthermore, femoral sections from these mice were subjected to TRAP staining, revealing no significant difference (Figure S3N). These findings collectively indicate that FGF9 may not substantially impact osteoclasts during OVX.

Altogether, the collective findings demonstrated that the loss-of-function mutation of *Fgf9* significantly reduced the OVX-induced bone loss and marrow adiposity. These results strongly suggest an essential role of *Fgf9* in maintaining the delicate balance between bone and fat in the skeleton.

### *Fgf9* inhibits osteogenic differentiation and promotes adipogenic differentiation of BMSCs *in vitro*

**Figure 3:**
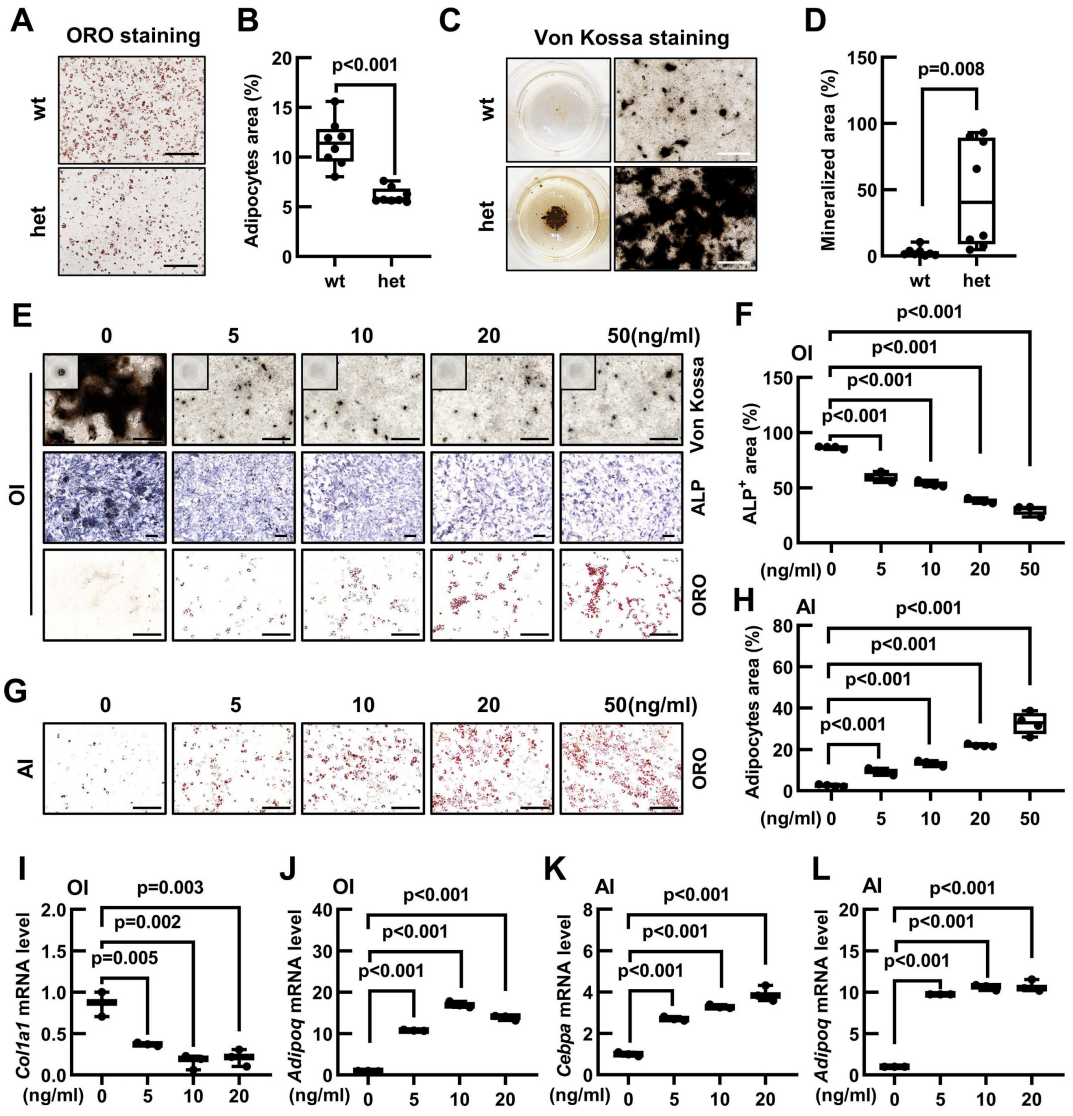
***Fgf9* inhibits osteoblastic differentiation and promotes adipogenic differentiation of BMSCs *in vitro*. (A-B)** Oil Red O (ORO) staining (A) and quantification analysis (B) showed the differentiated adipocytes in BMSCs from 20-month-old wt and het mice with adipogenic induction (AI) medium for 6 days. n = 8 mice in each group.** (C-D)** Von Kossa staining (C) and quantification analysis (D) showed the mineralized ECM generated by BMSCs from 20-month-old wt and het mice in osteogenic induced (OI) medium for 9 days. n = 8 mice in each group.** (E)** ALP, Von Kossa, and ORO staining of BMSCs under OI conditions with different concentrations of FGF9 stimulation (0, 5, 10, 20, and 50 ng/ml) for 9 days.** (F)** Quantification measurement of ALP-positive area percentage from (E), n = 4 independent experiments with biological replicates.** (G)** ORO staining of BMSCs under AI conditions with different concentrations of FGF9 stimulation (0, 5, 10, 20, and 50 ng/ml) for 6 days.** (H)** Quantification measurement of adipocyte area percentage from (G), n = 4 independent experiments with biological replicates.** (I-L)** BMSCs were cultured with different concentrations of FGF9 (0, 5, 10, and 20 ng/ml), and mRNA expression was detected by qRT-PCR. Relative mRNA levels of *Col1a1* (I) and *Adipoq* (J) in OI condition, and relative mRNA levels of *Cebpa* (K) and *Adipoq* (L) in AI condition, n = 3 biological replicates over three independent experiments. Data are analyzed by Student's t-test and shown as boxplots (median ± interquartile range). The BMSCs in (E-L) derived from 1-month-old wild-type mice. Scale bars represent 200 μm.

As a common progenitor of osteoblasts and adipocytes, the lineage commitment of BMSCs is crucial for maintaining the bone-fat balance. Our previous findings have revealed that *Fgf9^S99N^* mutation enhanced osteogenesis while concurrently inhibiting adipogenesis. Therefore, we have hypothesized that *Fgf9* can regulate the differentiation potential of BMSCs. The BMSCs were isolated from compact bones and identified using flow cytometry to detect cell surface markers for BMSC identification (negative markers: CD31, CD34, CD45, and Ter-119; positive markers: CD29, CD44, Sca-1, CD140a) (Figure S4A and B). BMSCs from 20-month-old wild-type and *Fgf9^wt/mut^* mice were differentiated into either adipocytes or osteoblasts. Oil Red O staining and quantitative analysis revealed that BMSCs isolated from *Fgf9^wt/mut^* mice had a diminished propensity to form matured adipocytes than that from wild-type mice (Figure 3A and B). In contrast, BMSCs derived from heterozygous mice exhibited a significant augmentation in the formation of mineralized nodules (Figure 3C and D). Further qRT-PCR analysis revealed that BMSCs derived from heterozygous mice had lower expression levels of adipogenic genes (*Cebpa*, *Pparg*, and *Adipoq*) and higher expression levels of osteogeneic genes (*Runx2*, *Osterix*, and *Alpl*) (Figure S4C and D).

Considering that the *Fgf9^S99N^* is a loss-of-function mutation, it is reasonable to speculate that *Fgf9* could inhibit osteogenesis while promoting adipogenesis in BMSCs. To substantiate this hypothesis, the 1-month-old wild-type BMSCs were isolated and cultured in an adipogenic or osteogenic induction medium containing different concentrations of recombinant FGF9 protein (0, 5, 10, 20, and 50 ng/ml). ALP staining, Von Kossa staining and quantitative assessment displayed that exogenous FGF9 significantly reduced osteoblastic differentiation and mineralization of BMSCs in a dose-dependent manner (Figure 3E and F; Figure S4E). Conversely, Oil Red O staining, coupled with quantitative analysis, demonstrated that FGF9 exerted a considerable promotive effect on adipogenesis of BMSCs in a dose-dependent manner (Figure 3G and H). Interestingly, it was also observed that even under osteogenic induction conditions, BMSCs were differentiated into adipocytes as FGF9 concentrations increased (Figure 3E; Figure S4F). To further consolidate these findings, qRT-PCR analysis revealed that FGF9 stimulation down-regulated the mRNA expression levels of osteogenic genes (*Runx2*, *Osterix*, and *Col1a1*) in BMSCs under osteogenic induction (Figure 3I; Figure S4I and J) and up-regulated the expression of adipogenic genes (*Cebpa*, *Pparg*, and *Adipoq*) under osteogenic and adipogenic conditions (Figure 3J-L; Figure S4G, H and K). Furthermore, FGF9 showed a similar effect on regulating osteogenic and adipogenic differentiation of BMSCs from rats (Figure S4L). These results underscore the role of *Fgf9* in promoting adipogenesis and inhibiting bone formation of BMSCs.

### *Fgf9* controls osteogenic/adipogenic differentiation of BMSCs *in vivo*

**Figure 4:**
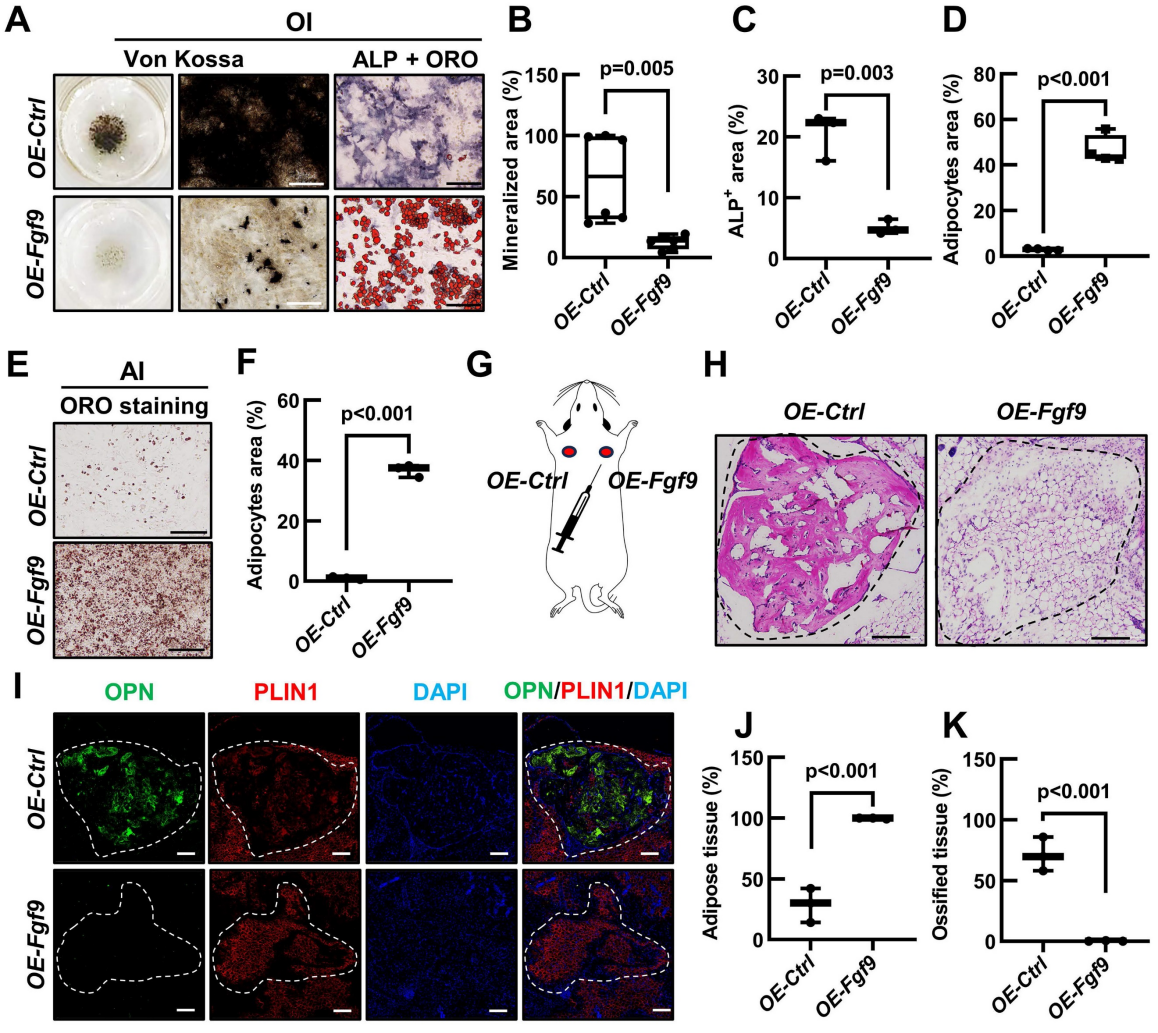
***Fgf9* controls osteogenic/adipogenic differentiation of BMSCs *in vivo*. (A)** *Fgf9* stable overexpressed (*OE-Fgf9*) and control (*OE-Ctrl*) BMSCs were differentiated with OI medium for 9 days. Von Kossa, ALP, ORO staining were performed.** (B-D)** Quantitative analysis of mineralization (B), ALP activity (C) and adipocytes area (D) from A. n ≥ 3 independent experiments with biological replicates**. (E-F)** ORO staining (E) and quantitative analysis (F) showed that *Fgf9* overexpression promoted BMSCs adipogenic differentiation under AI conditions for 6 days. n = 3 independent experiments with biological replicates. **(G)** A schematic diagram of subcutaneous injection of 5×10^5^* OE-Ctrl* and 5×10^5^ *OE-Fgf9* BMSCs into nude mice.** (H)** H&E staining images of *OE-Ctrl* and *OE-Fgf9* BMSCs implants after 5 weeks of injection.** (I)** Immunofluorescence staining of Osteopontin (OPN) and Perilipin A (PLIN1) in *OE-Ctrl* and *OE-Fgf9* BMSCs implants.** (J-K)** Statistical analysis of adipose tissue and ossified tissue percentages, n = 3 mice. Data are analyzed by Student's t-test and shown as boxplots (median ± interquartile range). Scale bars represent 200 μm.

To further elucidate the role of *Fgf9* in regulating the differentiation potential of BMSCs in an *in vivo* setting, we isolated BMSCs from 1-month-old wild-type mice and stably overexpressed *Fgf9* in BMSCs with lentivirus and antibiotic selection. qRT-PCR analysis showed a dramatic upregulation in *Fgf9* mRNA expression following transfection (Figure S5A). *In vitro* cytodifferentiation assay confirmed, again, that overexpressing *Fgf9* in BMSCs inhibited osteogenesis under osteogenic differentiation conditions (Figure 4A-C) and promoted adipogenesis under adipogenic and osteogenic differentiation conditions (Figure 4A and 4D-F). After the subcutaneous injection of *OE-Fgf9* and *OE-control* BMSCs into the scapular region of the same nude mouse (Figure 4G), the grafts were isolated and subjected to histological analysis after 5 weeks. H&E staining and quantitative assessment demonstrated that BMSCs in the control group had differentiated into both bone and adipose tissues, whereas *Fgf9*-overexpressed BMSCs had predominantly differentiated into adipose tissue (Figure 4H, J and K; Figure S5B). Immunofluorescence staining of OPN and PLIN1 further confirmed these observations (Figure 4I). These findings indicate that the overexpression of *Fgf9* significantly impedes osteogenic differentiation and promotes adipose differentiation of BMSCs both *in vivo* and *in vitro*.

### *Fgf9* controls the cell fate of BMSCs in the early stage of differentiation

**Figure 5:**
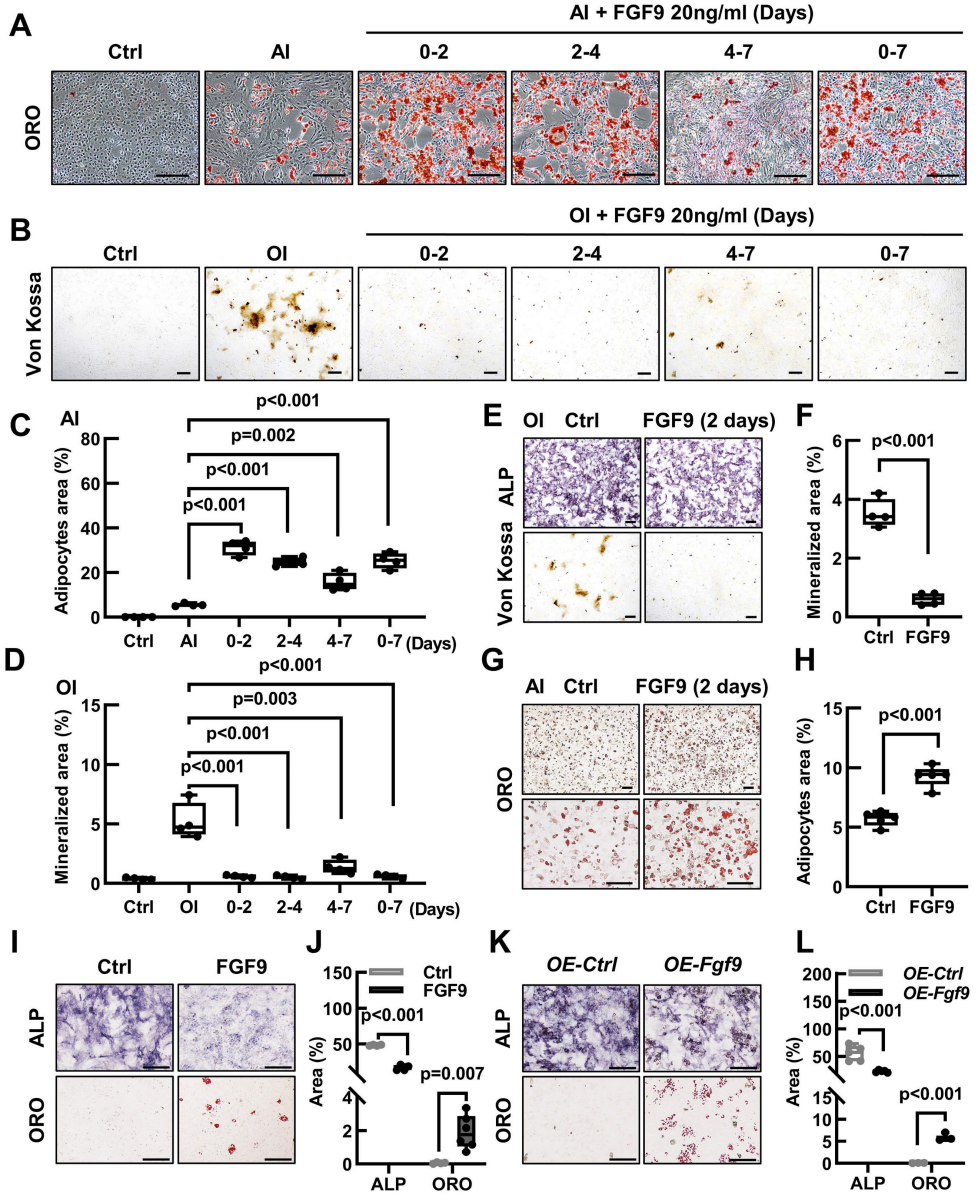
** FGF9 alters the osteogenic and adipogenic cell fate of BMSCs in the early stage of differentiation. (A)** BMSCs were cultured in an AI medium with 20 ng/ml recombinant FGF9 for the indicated periods. On day 7, ORO staining showed the adipocytes.** (B)** BMSCs were cultured in an OI medium with 20 ng/ml recombinant FGF9 for the indicated periods. On day 7, Von Kossa staining showed the mineralized ECM. Ctrl groups were maintained in the culture medium (CM).** (C-D)** Quantification measurement of adipocyte area percentage from (A) and mineralization area percentage from (B).** (E-H)** BMSCs were prior stimulated with FGF9 in the culture medium for 2 days, then differentiated with OI (E) and AI (G) medium. ALP, Von Kossa staining (E), and quantification analysis (F) indicated the osteogenic differentiation potential. ORO staining (G) and quantification analysis (H) indicated the adipogenic differentiation potential.** (I-J)** BMSCs were cultured in a culture medium for 9 days with or without 20 ng/ml FGF9. ALP and ORO staining (I) were performed and quantitatively analyzed (J).** (K-L)** *OE-Fgf9* and *OE-Ctrl* BMSCs were cultured in a culture medium for 9 days. ALP, ORO staining (K), and quantification analysis (L) were used to detect the spontaneous differentiation. n ≥ 3 independent experiments with biological replicates**.** Data are analyzed by Student's t-test and shown as boxplots (median ± interquartile range). Scale bars represent 200 μm.

The differentiation of BMSCs into osteoblasts and adipocytes follows a sequential process, delineated into three distinct stages: commitment, differentiation, and maturation 1. To investigate the effect of *Fgf9* on the differentiation of BMSCs, we stimulated them with FGF9 at various stages of differentiation. During the early stages of BMSCs differentiation, FGF9 stimulation was found to increase adipogenesis while decreasing osteogenesis, whether under osteogenic or adipogenic induction conditions (Figure 5A-D). Furthermore, the impact of FGF9 stimulation reduced gradually as the timing of the stimulation was delayed (Figure 5A-D). The results emphasized the significant role of FGF9 during the early stages of BMSCs differentiation, indicating its potential involvement in the fate determination of BMSCs. To further explore this, we pre-treated primary BMSCs with FGF9 for two days under standard culture conditions. After carefully washing to eliminate any residual FGF9, the BMSCs were induced for differentiation towards osteogenesis and adipogenesis *in vitro*. The differentiated results revealed that FGF9 pretreatment markedly suppressed osteogenic differentiation and mineralization while significantly promoting adipogenic differentiation (Figure 5E-H). Furthermore, prolonged stimulation of recombinant FGF9 to 8 days under culture conditions induced spontaneous differentiation of BMSCs into adipocytes and weakened ALP activity (Figure 5I and J). Moreover, BMSCs overexpressing *Fgf9* similarly exhibited adipogenic spontaneous differentiation (Figure 5K and L). These findings indicate that *Fgf9* is a potent cytokine that promotes adipogenic differentiation in BMSCs, particularly during the commitment phase.

### *Fgf9* alters the osteogenic and adipogenic gene expression in BMSCs

**Figure 6:**
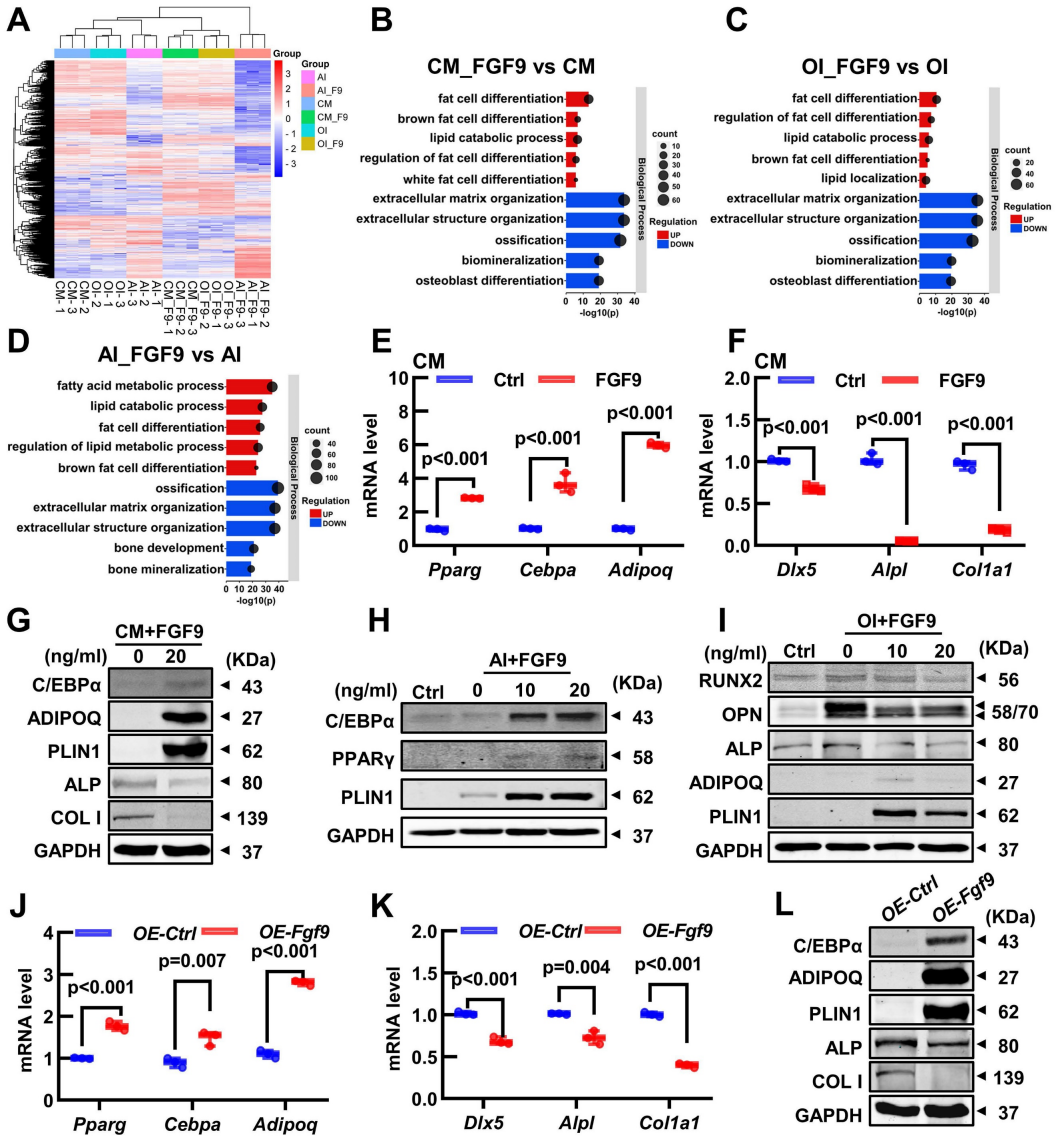
** FGF9 regulates BMSCs cell fate via changing the expression of osteoblastic and adipogenic genes. (A)** The heatmap of differentially expressed genes (DEGs) from 3 independent RNA-seq analyses in BMSCs with CM, OI, and AI conditions with/without 20 ng/ml FGF9 stimulation for 4 days.** (B-D)** Gene Ontology (GO) classification of DEGs from CM (B), OI (C), and AI (D) conditions. The adipogenesis and osteogenesis related terms were presented.** (E-F)** Relative mRNA level of adipogenic genes (*Pparg*, *Cebpa*, *Adipoq*) and osteogenic genes (*Dlx5*, *Alpl*, *Col1a1*) in BMSCs with FGF9 stimulation for 4 days in CM.** (G-I)** Protein levels of adipogenic genes (C/EBPα, PPARγ, ADIPOQ, PLIN1) and osteogenic genes (ALP, COL1, RUNX2, OPN) were detected by immunoblotting in BMSCs under CM (G), AI (H) and OI (I) conditions with/without FGF9 stimulation.** (J-K)** Relative mRNA level of *Pparg*, *Cebpa*, *Adipoq* (J), and *Dlx5*, *Alpl*, and *Col1a1* (K) in *OE-Ctrl* and *OE-Fgf9* BMSCs.** (L)** Protein levels of C/EBPα, ADIPOQ, PLIN1, ALP, and COL1 in *OE-Ctrl* and *OE-Fgf9* BMSCs were detected by immunoblotting. n = 3 biological replicates over three independent experiments. DEGs are defined as |Log_2_FC|≥1 and adjusted P-value≤0.05, corrected P-value of GO terms < 0.05. Data are analyzed by Student's t-test and shown as boxplots (median ± interquartile range).

To investigate the crucial mechanism, we conducted RNA-seq on 6 groups of BMSCs, which were cultured with or without 20 ng/ml FGF9 for 4 days in the culture medium (CM, CM_F9), osteogenic induction medium (OI, OI_F9), and adipogenic induction medium (AI, AI_F9). Volcano plots were used to show the differentially expressed genes (DEGs) in the FGF9-stimulated group, compared to the control group under different conditions (Figure S6A-C). Heatmap analysis of all DEGs demonstrated consistent patterns among the triplicate samples within each group (Figure 6A; Figure S6D-F). Following this, a Gene Ontology (GO) enrichment analysis of DEGs was performed to validate the differentiated status of BMSCs. Notably, enriched GO terms associated with adipogenesis and fat metabolism were significantly up-regulated in the CM, OI, and AI conditions with FGF9 stimulation. These terms included fat cell differentiation, regulation of fat cell differentiation, and lipid catabolic process. On the other hand, GO terms associated with bone formation such as extracellular matrix organization, ossification, biomineralization, and osteoblast differentiation, were found to be down-regulated (Figure 6B-D).

To assess the RNA-seq results, qRT-PCR and Western blotting were used to measure the gene transcription and protein expression levels of BMSCs. Under culture conditions, FGF9 stimulation and overexpression in BMSCs resulted in a significant decrease in the transcription of osteogenic genes (*Dlx5*, *Alpl*, and *Col1a1*) and protein expression (ALP and COL1) (Figure 6F, G, K and L, Figure S7B and G). At the same time, there was an increase in the transcription of adipogenic genes (*Cebpa*, *Pparg*, and *Adipoq*) and protein expression (C/EBPα, ADIPOQ, and PLIN1) (Figure 6E, G, J and L, Figure S7A and F). Similarly, under adipogenic and osteogenic differentiation, mRNA expression of osteogenic genes was significantly inhibited in BMSCs exposed to 20 ng/ml FGF9 (Figure S6H and J). In contrast, there was a marked increase in the mRNA level of adipogenesis genes under adipogenic induction (Figure S6I) and a modest increase under osteogenic induction (Figure S6G). Meanwhile, protein expression of BMSCs revealed that FGF9 increased adipogenesis (ADIPOQ and PLIN1) and decreased bone formation (RUNX2, OPN, and ALP) during osteogenesis induction (Figure 6I, Figure S7D and E), and significantly enhanced the expression of adipose-related genes (C/EBPα, PPARγ, and PLIN1) during adipogenesis induction (Figure 6H, Figure S7C). These findings suggest that *Fgf9* regulates the fate of BMSCs towards adipogenesis instead of osteogenesis by modulating gene transcription and expression.

### *Fgf9* activates multiple signaling pathways in BMSCs

**Figure 7:**
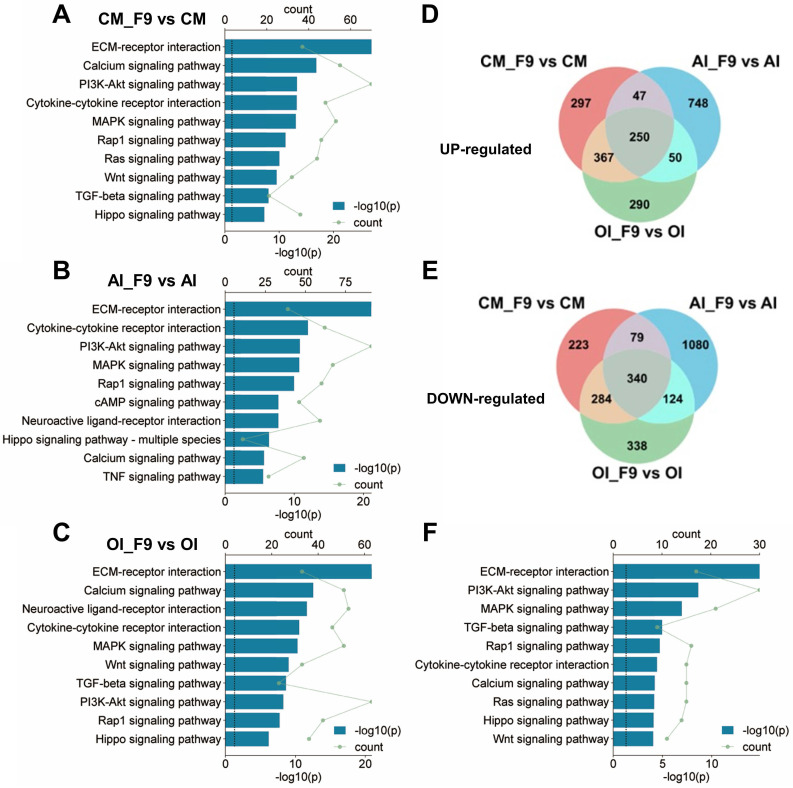
** FGF9 stimulation activates multiple signaling pathways in BMSCs. (A-C)** Kyoto Encyclopedia of Genes and Genomes (KEGG) enrichment of DEGs from CM (A), AI (B), and OI (C) groups. Bar diagrams showed the top 10 significant pathways for Environmental Information Processing.** (D-E)** Venn diagram revealed 250 co-up-regulated genes (D) and 340 co-down-regulated genes (E) in three compared groups of BMSCs.** (F)** KEGG enrichment of 590 co-regulated DEGs from D and E, and listed the top 10 significant pathways for Environmental Information Processing. DEGs are defined as |Log_2_FC|≥1 and adjusted P-value≤0.05, corrected P-value of pathways < 0.05.

In order to investigate the signaling cascades through which *Fgf9* modulates the differentiation of BMSCs, we employed Kyoto Encyclopedia of Genes and Genomes (KEGG) enrichment analysis on the DEGs enriched under the CM, OI, and AI conditions. The results revealed that FGF9 activated multiple signaling pathways within BMSCs under all three culture conditions, primarily through downstream signaling facilitated by FGFR, such as PI3K/AKT, MAPK, Rap1, and Ras signaling. Additionally, FGFR cross-talking signaling and other crucial signaling pathways, such as Wnt and TGF-beta, were activated (Figure 7A-C). It is worth noting, however, that the specific signaling pathways activated by FGF9 differed depending on the culture conditions used. For instance, the PI3K-AKT signaling pathway was more strongly activated in CM and AI conditions compared to the OI condition (Figure 7A-C).

To further discover the co-active pathways between CM, OI, and AI conditions, we intersected the up-regulated or down-regulated DEGs in each condition, resulting in a total of 250 co-up-regulated genes and 340 co-down-regulated genes (Figure 7D and E). Then, we performed KEGG analysis on the 590 co-regulated DEGs and unveiled the top 10 significant pathways associated with Environmental Information Processing. These pathways include ECM-receptor interaction, PI3K-Akt signaling pathway, MAPK signaling pathway, TGF-beta signaling pathway, Rap1 signaling pathway, Cytokine-cytokine receptor interaction, Calcium signaling pathway, Ras signaling pathway, Hippo signaling pathway, and Wnt signaling pathway (Figure 7F). Notably, these signaling pathways have been demonstrated to be associated with adipose differentiation and bone formation.

### *Fgf9* inhibits osteogenesis via MEK/ERK pathway and promotes adipogenesis via PI3K/AKT/Hippo pathway

**Figure 8:**
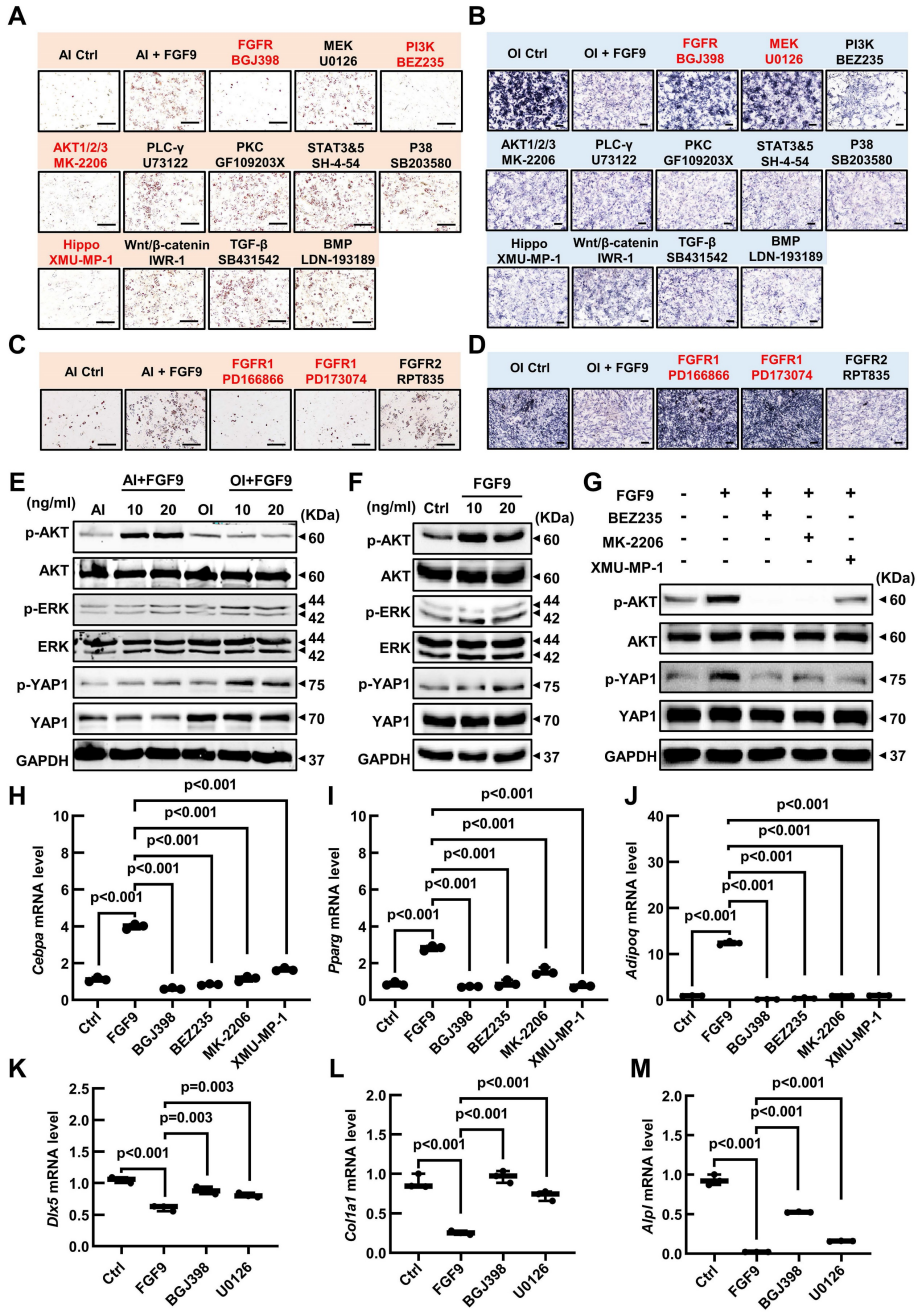
** FGF9 regulates BMSCs osteogenic/adipogenic fate through MEK/ERK, PI3K/AKT, and Hippo signaling pathways. (A)** ORO staining showed the effect of the indicated inhibitors on adipose differentiation of BMSCs with 20 ng/ml FGF9 stimulation in an AI medium. BMSCs were cultured for 6 days, the red-labeled inhibitors showed a significant effect.** (B)** ALP staining showed the effect of the indicated inhibitors on osteogenic differentiation of BMSCs with 20 ng/ml FGF9 stimulation in the OI medium. BMSCs were cultured for 6 days, the red-labeled inhibitors showed a significant effect.** (C-D)** ORO staining (C) and ALP staining (D) showed the effect of FGFR1 and FGFR2 inhibitors on the differentiation of BMSCs with 20 ng/ml FGF9 stimulation. BMSCs were cultured for 6 days, the red-labeled inhibitors showed a significant effect.** (E)** Immunoblotting analysis showed the phosphorylated and total protein levels of ERK, AKT, and YAP1 in BMSCs with FGF9 stimulation (0, 10, and 20 ng/ml) under AI or OI conditions for 6 days. **(F)** Immunoblotting analysis showed the phosphorylated and total protein levels of ERK, AKT, and YAP1 in the BMSCs stimulated with FGF9 (0, 10, and 20 ng/ml). BMSCs were cultured for 4 days under CM conditions.** (G)** Immunoblotting results showed the phosphorylated and total protein levels of AKT and YAP1 in BMSCs, which were pre-treated with inhibitors (BEZ235, MK-2206, and XMU-MP-1) for 10 hours and stimulated with 20 ng/ml FGF9 for 10min.** (H-J)** Relative mRNA levels of *Cebpa*, *Pparg*, and *Adipoq* in BMSCs stimulated with 20 ng/ml FGF9 and inhibitors (BGJ398, BEZ235, MK-2206, and XMU-MP-1) under CM conditions for 4 days.** (K-M)** Relative mRNA levels of *Dlx5*, *Alpl*, and *Col1a1* in BMSCs stimulated with 20ng/ml FGF9 and inhibitors (BGJ398 and U0126) under CM conditions for 4 days. n = 3 biological replicates over three independent experiments. Data are analyzed by Student's t-test and shown as boxplots (median ± interquartile range). Scale bars represent 200 μm.

Our research has found that *Fgf9* is closely associated with the FGFR signaling, TGF-beta, Hippo, and Wnt signaling pathways. To investigate the effect of FGF9 on the osteogenic and adipogenic differentiation of BMSCs, we used specific inhibitors for each of these signaling pathways. BMSCs were cultured in OI or AI medium containing 20 ng/ml FGF9, along with the indicated inhibitors (Table S1). Under adipogenic induction, inhibitors of FGFRs (BGJ398), PI3K (BEZ235), AKT (MK-2206) and Hippo (XMU-MP-1) markedly reversed the promoting effect of FGF9 on adipogenesis, while other inhibitors had little effect (Figure 8A; Figure S8A). Moreover, under osteogenic induction, ALP staining showed that only FGFR (BGJ398) and MEK (U0126) inhibitors significantly rescued the inhibitory effect of FGF9 on osteogenesis, and other inhibitors had no effect (Figure 8B; Figure S8B). In order to further clarify the role of FGF9 in regulating BMSCs differentiation through which FGFR, we initially examined the expression levels of *Fgfr1-4* in BMSCs using qRT-PCR. The results showed that *Fgfr1* and *Fgfr2* were predominantly expressed in BMSCs (Figure S8C). Interestingly, upon stimulation with FGF9, there was a significant increase in the mRNA expression level of *Fgfr1*, whereas the expression level of *Fgfr2* notably decreased (Figure S8D), suggesting that *Fgfr1* may play a more important role in FGF9-regulated BMSCs differentiation. Subsequently, we treated BMSCs with specific inhibitors of FGFR1 and FGFR2 during the differentiation processes towards osteogenesis and adipogenesis with 20ng/ml FGF9, respectively 49-51. The results demonstrated that FGFR1 inhibitors (PD166866 and PD173074) significantly inhibited the effects of FGF9 on promoting adipogenesis and inhibiting osteogenesis, while the specific inhibitor of FGFR2 (RPT835) had almost no effect (Figure 8C and D; Figure S8E and F). These findings indicate that FGF9 primarily regulates BMSCs differentiation through FGFR1.

To further elucidate the downstream signaling pathways activated by FGF9, western blotting analysis revealed that FGF9 stimulation increased p-AKT and p-YAP1 in BMSCs under adipogenesis induction, and enhanced p-ERK and p-YAP1 levels in osteogenic conditions (Figure 8E; Figure S9A and B). Considering the important role of FGF9 in early fate decisions, we stimulated BMSCs with 10 and 20 ng/ml FGF9 under culture conditions. The stimulation of FGF9 enhanced the phosphorylation levels of ERK, AKT, and YAP1 (Figure 8F; Figure S9C). To further evaluate the effects of FGF9 and specific inhibitors on osteogenesis and adipogenesis, we analyzed the expression levels of adipogenic and osteogenic genes in BMSCs under culture conditions. BGJ398, PD166866, PD173074, BEZ235, MK-2206, and XMU-MP-1 inhibitors markedly reversed the promoting effect of FGF9 on the mRNA levels of *Cebpa*, *Pparg*, and *Adipoq* (Figure 8H-J; Figure S8H-J), and BGJ398, PD166866, PD173074 and U0126 inhibitors significantly rescued the inhibitory effect of FGF9 on *Dlx5*, *Alpl*, and *Col1a1* (Figure 8K-M; Figure S8K-M). These results demonstrated that *Fgf9* inhibits osteogenesis mainly via the MEK/ERK pathway and facilitates adipogenesis via the PI3K/AKT and Hippo pathways.

Hippo signaling is an evolutionarily conserved pathway that controls cell proliferation, differentiation, apoptosis, and stem cell self-renewal 52. Research has indicated that the RTK signaling regulates the Hippo pathway indirectly through downstream effectors such as PI3A/AKT and MAPK in tumor cells 53. The BMSCs differentiation experiment revealed that the inhibitor of the Hippo pathway, XMU-MP-1, has a specific effect only under the AI conditions. On the other hand, FGF9 primarily activates the PI3K/AKT signaling pathway under AI conditions. Based on these findings, we have developed a hypothesis that PI3A/AKT may work as an upstream regulator of the Hippo pathway. Thus, we stimulated BMSCs with a brief exposure of 20 ng/ml FGF9 for different durations (0, 5 min, 10 min, and 30 min), revealing a rapid increase of p-AKT and p-ERK at 5 minutes (Figure S8G; Figure S9D). However, the p-ERK started to decline after 10 minutes of stimulation (Figure S8G; Figure S9D). Interestingly, the phosphorylation of YAP1 exhibited a noteworthy escalation at 10 minutes, which occurred after the phosphorylation of AKT (Figure S8G; Figure S9D). To further validate this hypothesis, we pre-treated BMSCs with inhibitors of the PI3K/AKT signaling pathway (BEZ235, MK-2206) for 10 hours, followed by a 10-minute stimulation with 20 ng/ml FGF9. Western blotting results revealed that PI3K (BEZ235) and AKT (MK-2206) inhibitor attenuated the FGF9-induced phosphorylation of YAP1, which aligned with the effects observed with the Hippo pathway inhibitor (XMU-MP-1) (Figure 8G; Figure S9E).

In summary, these findings demonstrate that FGF9 suppresses the osteogenic differentiation and mineralization of BMSCs via FGFR1 by activating the MEK/ERK signaling pathway and promotes adipogenesis by activating the PI3K/AKT/Hippo signaling pathway.

## Discussion

In this study, we identified *Fgf9* as a bone marrow microenvironment growth factor that regulated the balance between bone and adipose tissue in adult bone. Using *Fgf9* loss-of-function mutation in mice, the formation of BMAT was notably diminished in both adult mice and mice induced with ovariectomy while concurrently promoting substantial bone formation. This regulatory impact was accompanied by a modification in the differentiation potential of BMSCs, shifting from osteogenesis to adipogenesis upon stimulation by FGF9, especially during the cell commitment stage of BMSCs differentiation. FGF9 stimulation significantly inhibited osteogenic gene expression and promoted adipogenic gene expression, regardless of the differentiaiton status of BMSCs. Mechanistic investigations revealed that FGF9 repressed the osteogenesis of BMSCs by activating the MEK/ERK signaling pathway and promoted adipogenesis by activating the PI3K/AKT/Hippo signaling pathway.

FGF9 is a pivotal cytokine regulating skeletal development but has a critical negative regulatory role in maintaining adult bone homeostasis. During bone development, *Fgf9* promotes bone formation by promoting cell proliferation and vascularization. *Fgf9^-/-^* mice exhibit reduced blood vessel formation in long bones, leading to delayed mineralization center formation and ultimately resulting in shortened skeletal segments 31. Fakhry *et al.* highlight that FGF9 stimulates the proliferation of osteoblasts derived from cranial bones 40. Furthermore, overexpressing *Fgf9* in cranial mesenchymal cells significantly facilitates the proliferation of mesenchymal cells 54. Behr *et al.* prove that *Fgf9* is indispensable for bone repair by enhancing bone formation and vascularization 55. Several *in vitro* studies also demonstrate that *Fgf9* promotes the proliferation of mesenchymal stem cells from bone marrow or other tissues 40, 41, 56, 57. However, unlike the rapid cell proliferation, differentiation, and morphogenesis observed during development or injury repair in the skeleton, mesenchymal stem cells in the adult bone exist in a low-proliferation state and sustain an appropriate differentiation status, which is crucial for maintaining the bone homeostasis 1. Numerous studies across different species have revealed that FGF9 inhibits mesenchymal stem cell/osteoprogenitor cell differentiation and mineralization. Lu J. *et al.* demonstrate that continuously stimulating cells with FGF9 protein inhibits osteogenesis and extracellular mineralization in BMSCs, DPSCs, and calvaria-derived mesenchymal cells 39, 41. Similarly, Fakhry *et al.* reveal that continuous treatment of osteoblasts with FGF9 suppresses osteogenesis and mineralization 40. In our previous research, we have reported the inhibitory effect of *Fgf9* on osteogenic differentiation and promotion of osteoclast differentiation and bone resorption, thereby exerting a negative regulatory role on bone density 38. This study further revealed the capability of *Fgf9* to induce a transformation of the cell fate of BMSCs from osteoblast to adipocyte, promoting the formation of BMAT and inhibiting bone formation. Interestingly, the effects of *Fgf9* on bone homeostasis are in line with the pathological manifestation characterized by a reduction in bone formation and an increased accumulation of BMAT in senile osteoporosis 58. The expression level of *Fgf9* was alleviated in the aged and OVX-induce bone and fibroblasts. These observations led us to speculate that *Fgf9*, as a negative regulator of bone homeostasis, may play a crucial role in the development of senile osteoporosis. To investigate this further, it is necessary to study the *in vivo* regulatory roles of *Fgf9* in adult bone using *Fgf9*-inducible conditional overexpression or knockout mouse models. More importantly, it is necessary to confirm the expression levels of *FGF9* in correlation with the progression of osteoporosis in clinical studies involving elderly individuals and patients with senile osteoporosis.

BMSCs with self-renewability and multipotency are thought to give rise to bone marrow preadipocyte-like and osteoprogenitor cells and differentiate into terminal osteoblasts or adipocytes 17, 59. This complex process of BMSCs fate determination is tightly controlled via several secreted cytokines and transcription factors, possibly in a sequential cascade 60. Our research indicates that the impact of *Fgf9* on BMSCs differentiation primarily occurs during the fate determination stage. However, increasing evidence suggests that BMSCs are a heterogeneous cell population of stem cells with different differentiation potentials 8, 61. Ambrosi *et al.* identify osteogenic progenitor cells (OPCs) capable of osteoblastic/chondrogenic differentiation, adipogenic progenitor cells (APCs) capable of adipogenic differentiation, and tri-lineage progenitor cells (TPCs) within BMSCs 8. Moreover, single-cell transcriptomic studies have further subdivided the BMSCs population into more refined and diverse subpopulations 25, 26, 62, 63. Thus, the regulation of FGF9 on BMSCs differentiation may not only influence the lineage commitment of the entire cell population but also change the proportions of different fate-determined subpopulations. For instance, FGF9 might exhibit differential effects on the proliferation of distinct subgroups of stem cells since the *Fgfrs* expression patterns are cell type-dependent 64. It is crucial to employ techniques such as single-cell RNA sequencing and lineage tracing to elucidate the underlying mechanisms for detailed investigation.

The binding of FGF9 to FGFR leads to receptor dimerization, resulting in reciprocal phosphorylation of the intracellular domain of FGFR and activation of downstream signaling pathways, including MAPK, PI3K/AKT, and PLCγ 19. Our findings indicate that FGF9 activates different signaling pathways to regulate BMSCs differentiation under different conditions via FGFR1. This suggests that BMSCs exhibit distinct responses to FGF9 stimulation in various environmental conditions or differentiation states, and the molecular mechanisms are not yet fully understood. One possible explanation is that BMSCs express different FGFRs in different statuses. *Fgfr1* and *Fgfr2* are the two major receptors predominantly expressed in BMSCs, and their expression profiles may vary across different stages of cell differentiation. For instance, previous studies have shown that *Fgfr1* is mainly expressed in osteoprogenitor cells, while *Fgfr2* is primarily expressed in mature osteoblasts 40. The mRNA expression of *Fgfr1* and *Fgfr2* in mesenchymal stem cells (MSCs) increased during adipocyte differentiation and osteoblast differentiation, respectively 65. Our previous research revealed that *Fgfr1* is upregulated while *Fgfr2* is downregulated in response to FGF9 stimulation in BMSCs 38. Besides, *Fgfr1* and *Fgfr2* play distinct roles in regulating the osteogenic and adipogenic differentiation of BMSCs. *Fgfr1* is primarily thought to facilitate the proliferation of MSCs 66, and its role in osteogenesis is controversial in the current literature. *Fgfr1* is a negative regulator of long bone growth, lacking *Fgfr1* in adult mice shows increased bone mass 64. However, deletion of *Fgfr1* led to delayed osteogenic differentiation in osteoprogenitor cells and accelerated osteogenesis in differentiated osteoblasts 67. On the other hand, some studies demonstrate that *Fgfr1* promotes adipogenic differentiation of adipose-derived stem cells (ASCs) and MSCs 65, 68. *Fgfr2* has been shown to positively regulate bone formation and promote osteogenic differentiation of MSCs 69, 70. Meanwhile, some researchers propose that *Fgfr2* inhibits adipose differentiation in MSCs and is pivotal in mediating the bone fat balance 71, 72. Thus, the disparity in expression and functional distinctions of *Fgfr1* and *Fgfr2* within BMSCs is one of the reasons behind the activation of distinct signaling pathways by FGF9 under adipogenic and osteogenic induction conditions. Another potential reason is that BMSCs exhibit different intracellular signaling backgrounds under different conditions. Even if FGF9 activates the same receptor, the crosstalk between FGFR and other signaling may contribute to preferences in activating specific signaling pathways under different conditions. In the future, it will be necessary to use *Fgfr1* and *Fgfr2* gene knockout or mutant mouse models to explore this phenomenon.

In summary, this study demonstrates that *Fgf9* plays a critical role in BMSCs fate decisions by facilitating adipogenesis and inhibiting osteogenesis. Our results indicate that *Fgf9* is involved in bone-fat imbalance of osteoporosis, which may be a new therapeutic strategy for patients with osteoporosis.

## Supplementary Material

Supplementary figures and tables.

## Abbreviations

BMSCs: bone marrow mesenchymal stem cells
FGF9: fibroblast growth factor 9
BMAT: bone marrow adipose tissue
GO: Gene Ontology
KEGG: Kyoto Encyclopedia of Genes and Genomes
H&E: Hematoxylin and eosin
qRT-PCR: quantitative reverse transcription PCR
WT: wild-type
AI: adipogenic induction
OI: osteogenic induction
CM: culture medium
OVX: ovariectomy
