# Supplementary material for: Fgf9 regulates bone marrow mesenchymal stem cell fate and bone-fat balance in osteoporosis by PI3K/AKT/Hippo and MEK/ERK signaling
Source: Int J Biol Sci. 2024 Jun 17;20(9):3461–79. doi: 10.7150/ijbs.94863 (PMC11234224; doi:10.7150/ijbs.94863)
Supplement: Supplementary file 1 — Supplementary figures and tables. [file ijbsv20p3461s1.pdf]

Supplementary Materials

Fgf9 regulates bone marrow mesenchymal stem cell fate and bone-fat balance in osteoporosis by PI3K/AKT/Hippo and MEK/ERK signaling

Mingmei Chen[1], Hui Liang[1], Min Wu[2], Haoyang Ge[1], Yan Ma[3], Yan Shen[1], Shunyuan Lu[1], Chunling Shen[1], Hongxin Zhang[1], Zhugang Wang[1*], Lingyun Tang[1*]

Table of Contents

Figure S1. *Fgf9* in bone marrow clusters.

Figure S2. *Fgf9*^{S99N} mutation mitigates BMAT accumulation in adult mice.

Figure S3. *Fgf9*^{S99N} mutation involves in bone-fat balance.

Figure S4. *Fgf9* regulates BMSCs differentiation both in mice and rat.

Figure S5. Differentiation of *OE-Ctrl*/*OE-Fgf9* BMSCs in vivo.

Figure S6. Analysis of DEGs and mRNA level of bone-fat related genes.

Figure S7. Quantitative results of protein levels from Figure 6.

Figure S8. *Fgf9* regulates BMSCs through FGFR1, PI3K/AKT and Hippo pathways.

Figure S9. Quantitative results of phosphorylated protein levels from Figure 8.

Table S1. Inhibitors used for signaling transduction.

Table S2. Primers used for mRNA expression detection and genotyping.

Table S3. Antibodies information.

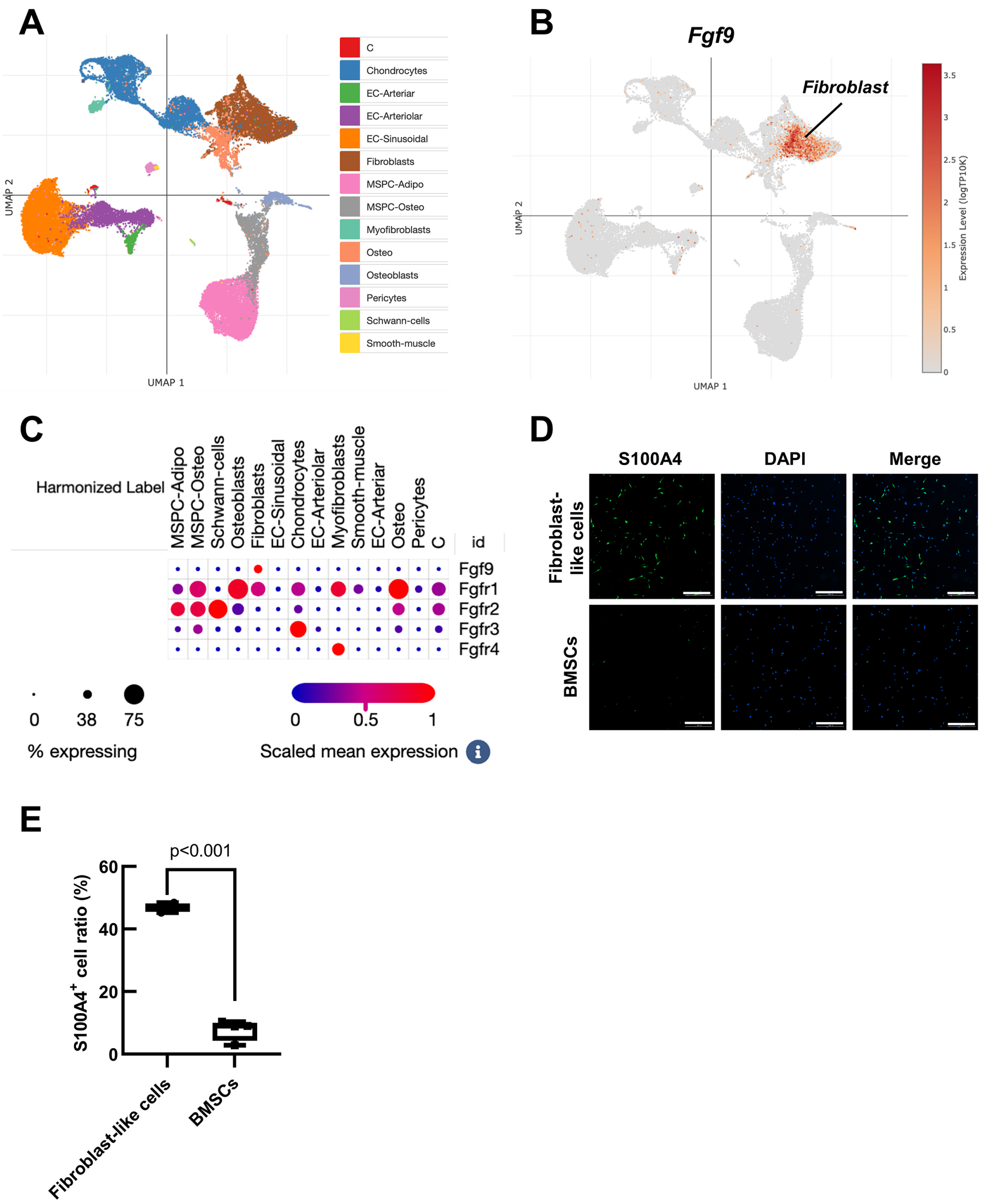

Figure S1. *Fgf9* in bone marrow clusters. (A) UMAP visualization of all bone marrow cells from the integrated analysis, color-coded based on the clusters. **(B)** Distribution of *Fgf9* in bone marrow stroma cell clusters. **(C)** Expression of *Fgf9* and *Fgfrs* in bone marrow clusters. The size of dots represents the percentage of expression; red and blue represent the level of scaled mean expression. **(D)** Immunofluorescence staining of S100A4 in Fibroblast-like cells and BMSCs. **(E)** S100A4$^+$ cell ratio in Fibroblast-like cells and BMSCs. Data are analyzed by Student's t-test and shown as boxplots (median ± interquartile range). Scale bars represent 300 μm.

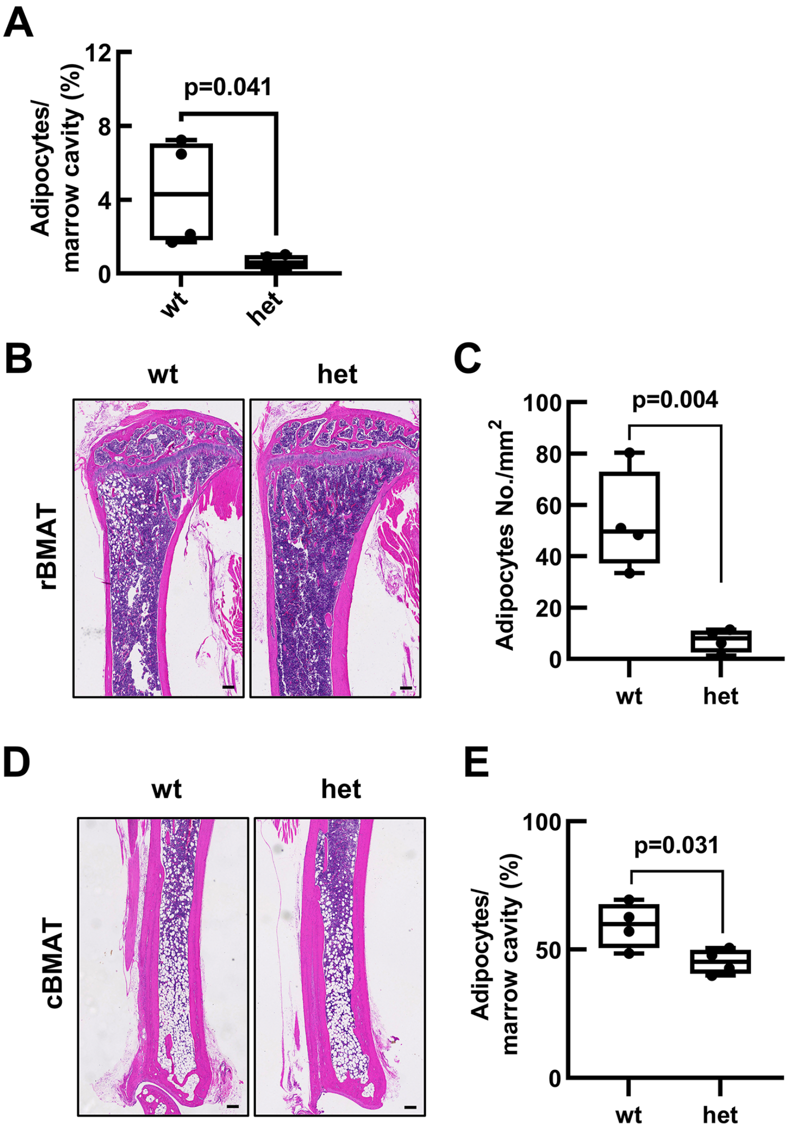

Figure S2. *Fgf9*^{S99N} mutation mitigates BMAT accumulation in adult mice. (A) Statistical analysis of femur adipocytes area in 4-month-old wt and het mice. **(B)** H&E staining of rBMAT in tibiae from 4-month-old male wild-type and heterozygous mice. **(C)** Statistical analysis of rBMAT adipocyte number in 4-month-old wt and het mice. **(D)** H&E staining of cBMAT in tibiae from 4-month-old male wt and het mice. **(E)** Statistical analysis of cBMAT adipocytes area in 4-month-old wt and het mice. Data are analyzed by Student's t-test and shown as boxplots (median ± interquartile range), n = 4 mice in each group. Scale bars represent 200 μm.

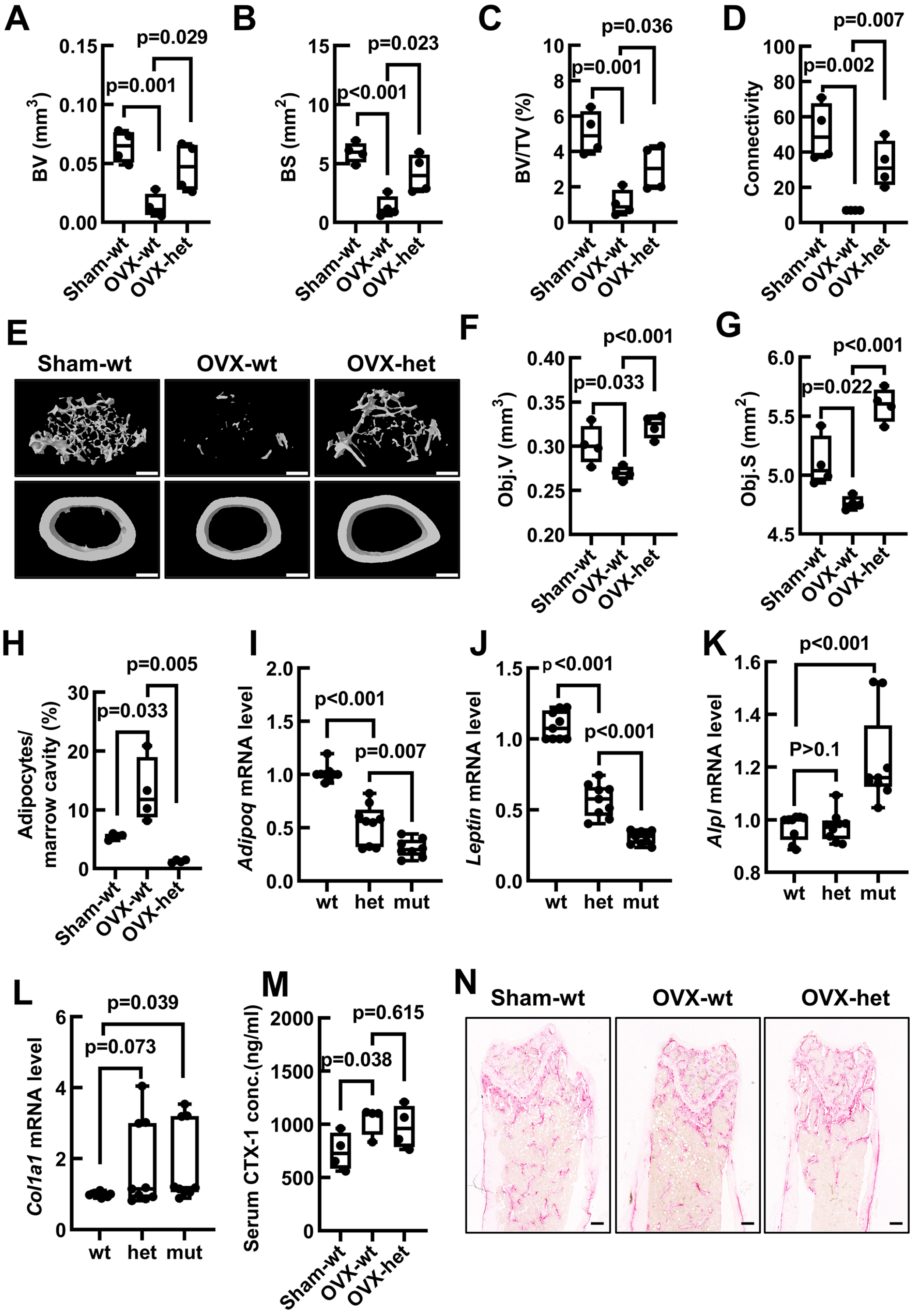

Figure S3. ***Fgf9**^{S99N}* **mutation involves in bone-fat balance. (A-D)** Bone volume (BV), Bone surface (BS), Percent bone volume (BV/TV), and Connectivity (Conn) were determined by micro-CT analysis. **(E)** Micro-CT images of the lateral-view of trabecular bone of the metaphyseal region (above) and top-view cortical bone (below),

scale bars represent 1 mm. **(F, G)** Cortical bone parameters were measured including Object volume (Obj.V) and Object surface (Obj.S). **(H)** Quantification analysis of femur adipocytes area / bone marrow cavity area percentage in Sham-wt, OVX-wt, and OVX-het mice. **(I-L)** Relative mRNA level of *Adipoq*, *Leptin*, *Alpl*, and *Col1a1* in femurs of neonatal wt, het, and homozygous (mut) mice. **(M)** Quantification analysis of serum CTX-1 level in Sham-wt, OVX-wt, and OVX-het mice by ELISA. **(N)** Representative images of TRAP staining in femurs of Sham-wt, OVX-wt, and OVX-het mice, scale bars represent 200 μm. Data are analyzed by Student's t-test and shown as boxplots (median ± interquartile range). In (A) - (H), (M), and (N), n = 4 in each group. In (I) - (L), n = 3 mice with 3 biological replicates in each group.

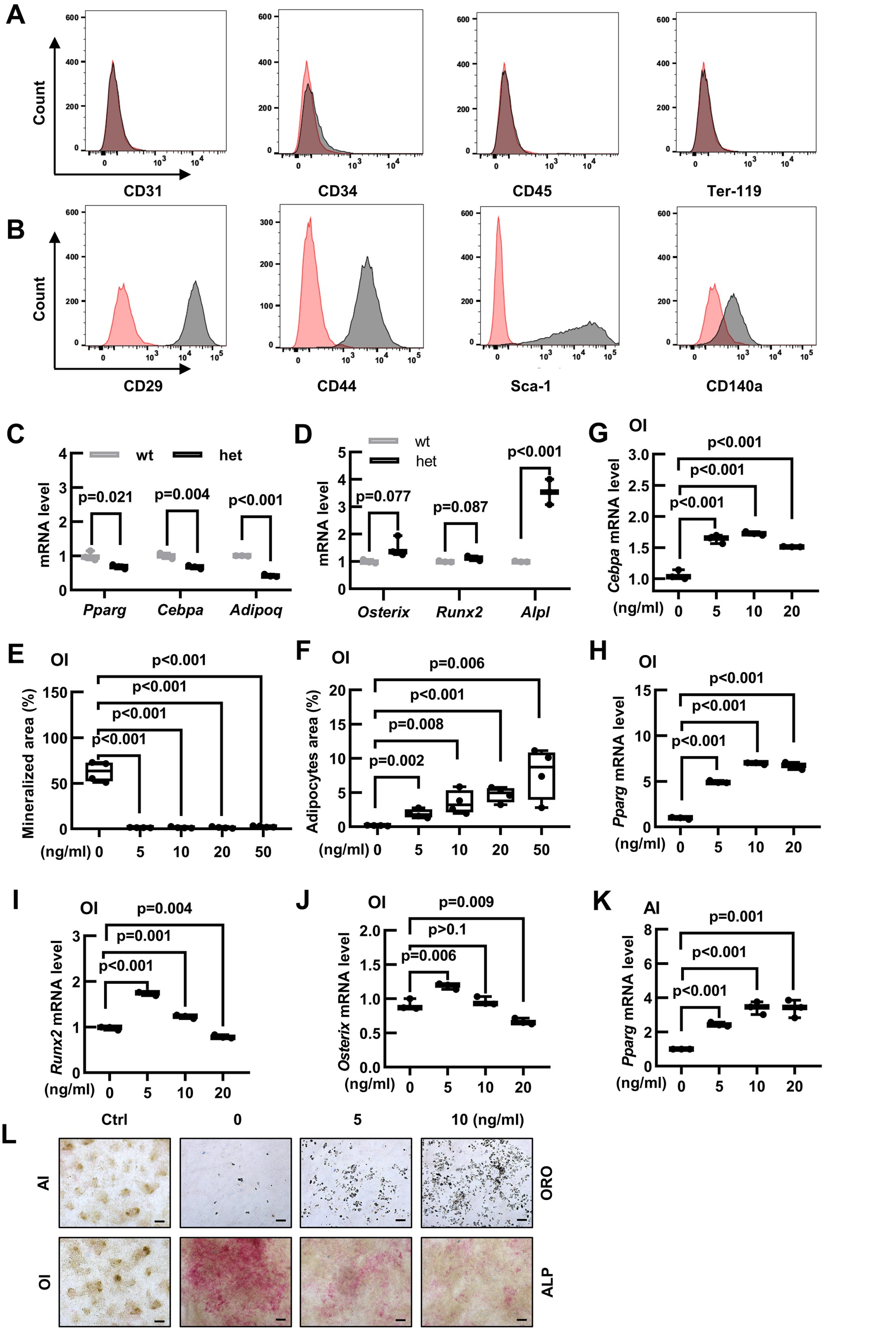

Figure S4. *Fgf9* regulates BMSCs differentiation both in mice and rat. (**A**, B) Negative surface markers (CD31, CD34, CD45, and Ter-119) (A) and positive markers (CD29, CD44, Sca-1, and CD140a) (B) of BMSCs were characterized using flow

cytometry. Isomorphic antibodies used as negative control. **(C, D)** Relative mRNA levels of *Cebpa*, *Pparg*, and *Adipoq* (C), *Osterix*, *Runx2*, and *Alpl* (D) in BMSCs from 20-month-old wt and het mice. **(E-F)** Quantification measurement of mineralization area and adipocytes area under OI condition. n = 4 independent experiments with biological replicates. **(G-J)** Relative mRNA levels of *Runx2*, *Osterix*, *Cebpa*, and *Pparg* in osteogenesis induction, while BMSCs were cultured with different concentrations of FGF9 (0, 5, 10 and 20 ng/ml). n = 3 biological replicates over three independent experiments. **(K)** Under adipogenesis induction, relative mRNA level of *Pparg* in BMSCs with different FGF9 concentrations (0, 5, 10 and 20 ng/ml). n = 3 biological replicates over three independent experiments. **(L)** ALP staining and Oil Red O staining of rat BMSCs in OI and AI medium with different concentrations of FGF9 (0, 5 and 10 ng/ml). Data are analyzed by Student's t-test and shown as boxplots (median ± interquartile range). Scale bars represent 200 μm.

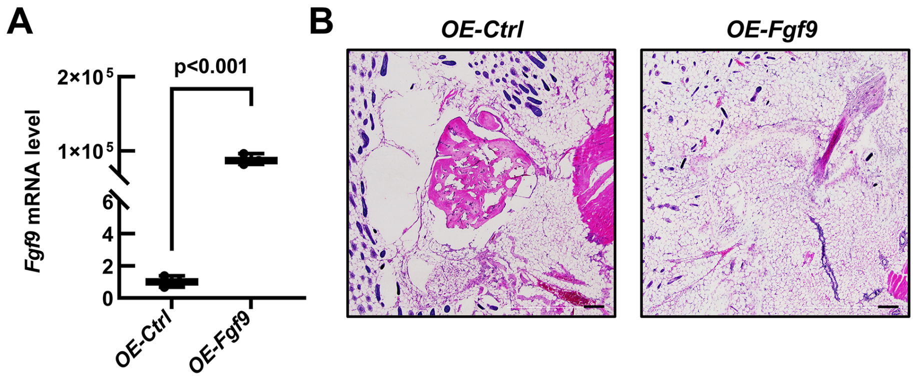

Figure S5. Differentiation of *OE-Ctrl*/*OE-Fgf9* BMSCs in vivo. (A) mRNA expression level of *Fgf9* increased dramatically after transfection. n = 3 biological replicates over three independent experiments. **(B)** After subcutaneous injection for 5 weeks, smaller magnifications of H&E staining, n = 3 mice. Data are analyzed by Student's t-test and shown as boxplots (median ± interquartile range). Scale bars represent 200 μm.

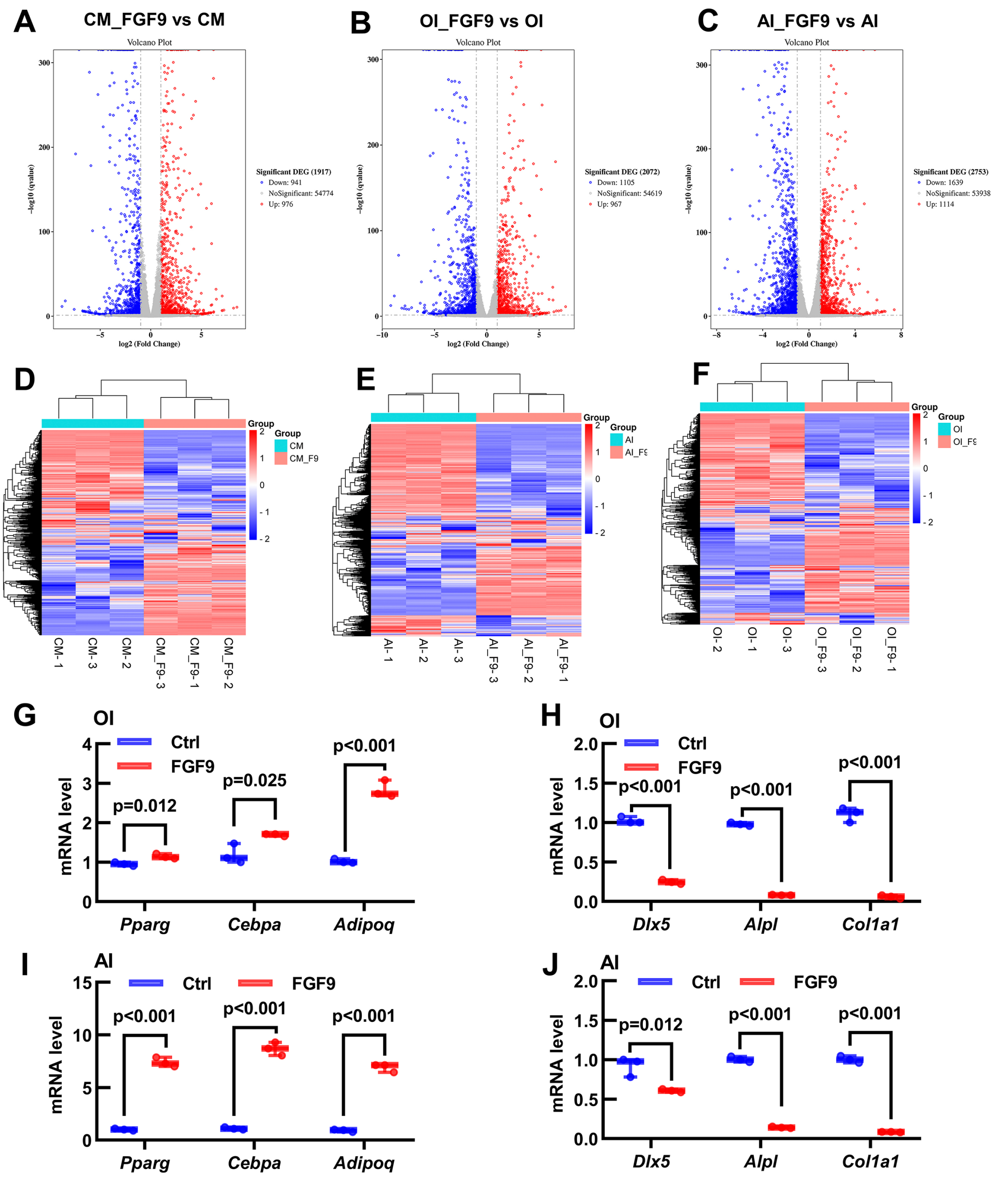

Figure S6. Analysis of DEGs and mRNA level of bone-fat related genes. (A-C) Volcano plots exhibited significantly DEGs under CM (976 up-regulated and 941 down-regulated genes) (A), OI (967 up-regulated and 1105 down-regulated genes) (B) and AI (1114 up-regulated and 1639 down-regulated genes) (C) conditions. **(D-F)** Heatmaps of DEGs showed changes between control and experimental groups under CM (D), AI (E) and OI (F) conditions. **(G-H)** Relative mRNA level of *Pparg*, *Cebpa*, *Adipoq* (G), and *Dlx5*, *Alpl*, and *Col1a1* (H) in BMSCs cultured with FGF9 under OI conditions. **(I-J)** Relative mRNA level of *Pparg*, *Cebpa*, *Adipoq* (I), and *Dlx5*, *Alpl*, and *Col1a1* (J) in BMSCs cultured with FGF9 under AI conditions. n = 3 biological

replicates over three independent experiments. DEGs are defined as $|Log_2FC| \geqslant 1$ and adjusted P-value$\leqslant$0.05. Data are analyzed by Student's t-test and shown as boxplots (median ± interquartile range).

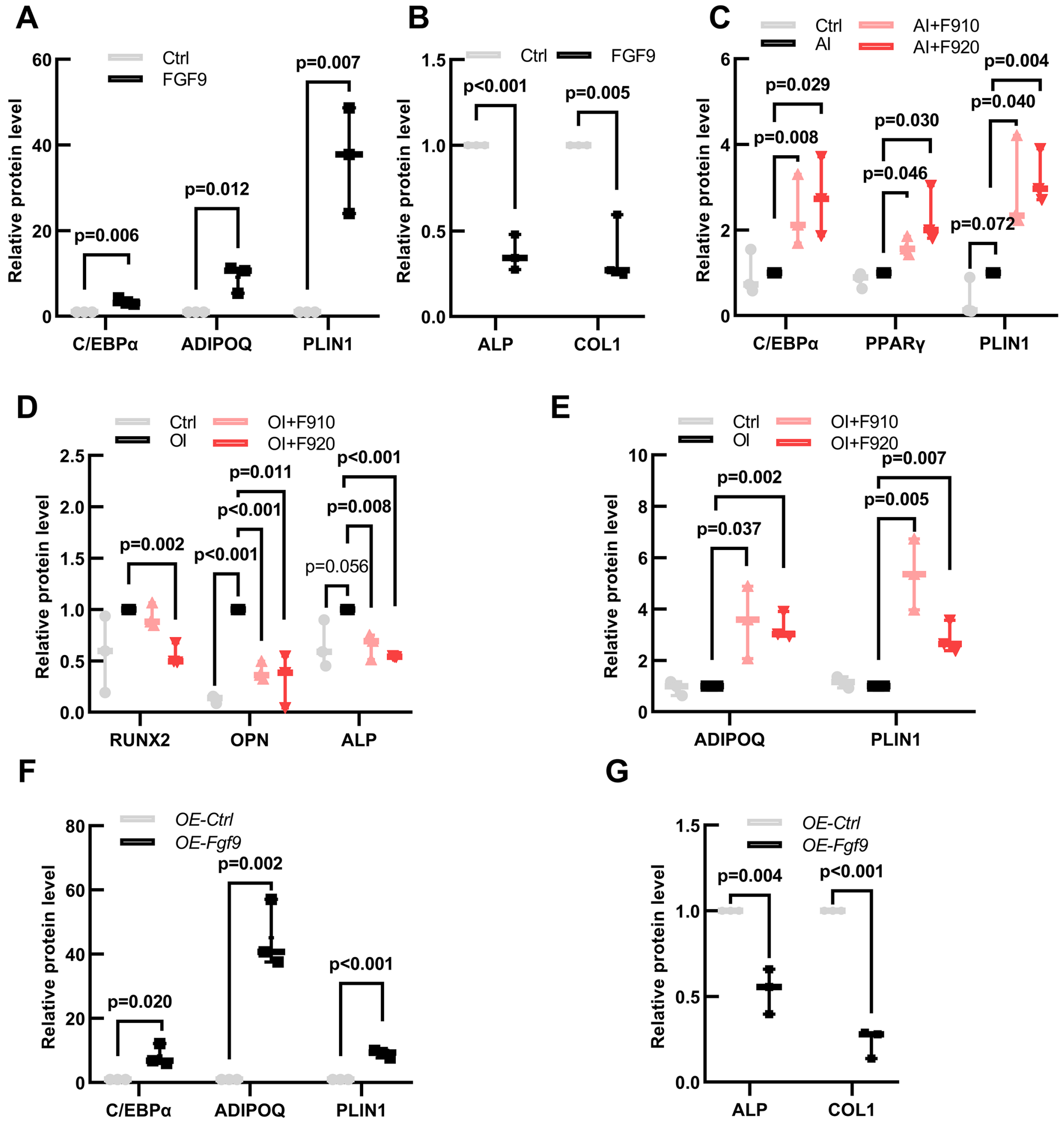

Figure S7. Quantitative results of protein levels from Figure 6. (A-B) Protein levels of adipogenic genes (C/EBPα, ADIPOQ, PLIN1) and osteogenic genes (ALP, COL1) were quantitatively analyzed in BMSCs under CM conditions with/without FGF9 stimulation referring to Figure 6G. **(C)** Protein levels of adipogenic genes (C/EBPα, PPARγ, PLIN1) were quantitatively analyzed in BMSCs under AI conditions with/without FGF9 stimulation referring to Figure 6H. **(D-E)** Protein levels of

osteogenic genes (RUNX2, OPN, ALP) and adipogenic genes (ADIPOQ, PLIN1) were quantitatively analyzed in BMSCs under OI conditions with/without FGF9 stimulation referring to Figure 6I. **(F-G)** Protein levels of C/EBPα, ADIPOQ, PLIN1, ALP, and COL1 in *OE-Ctrl* and *OE-Fgf9* BMSCs were quantitatively measured referring to Figure 6L. n = 3 biological replicates over three independent experiments. Data are analyzed by Student's t-test and shown as boxplots (median ± interquartile range).

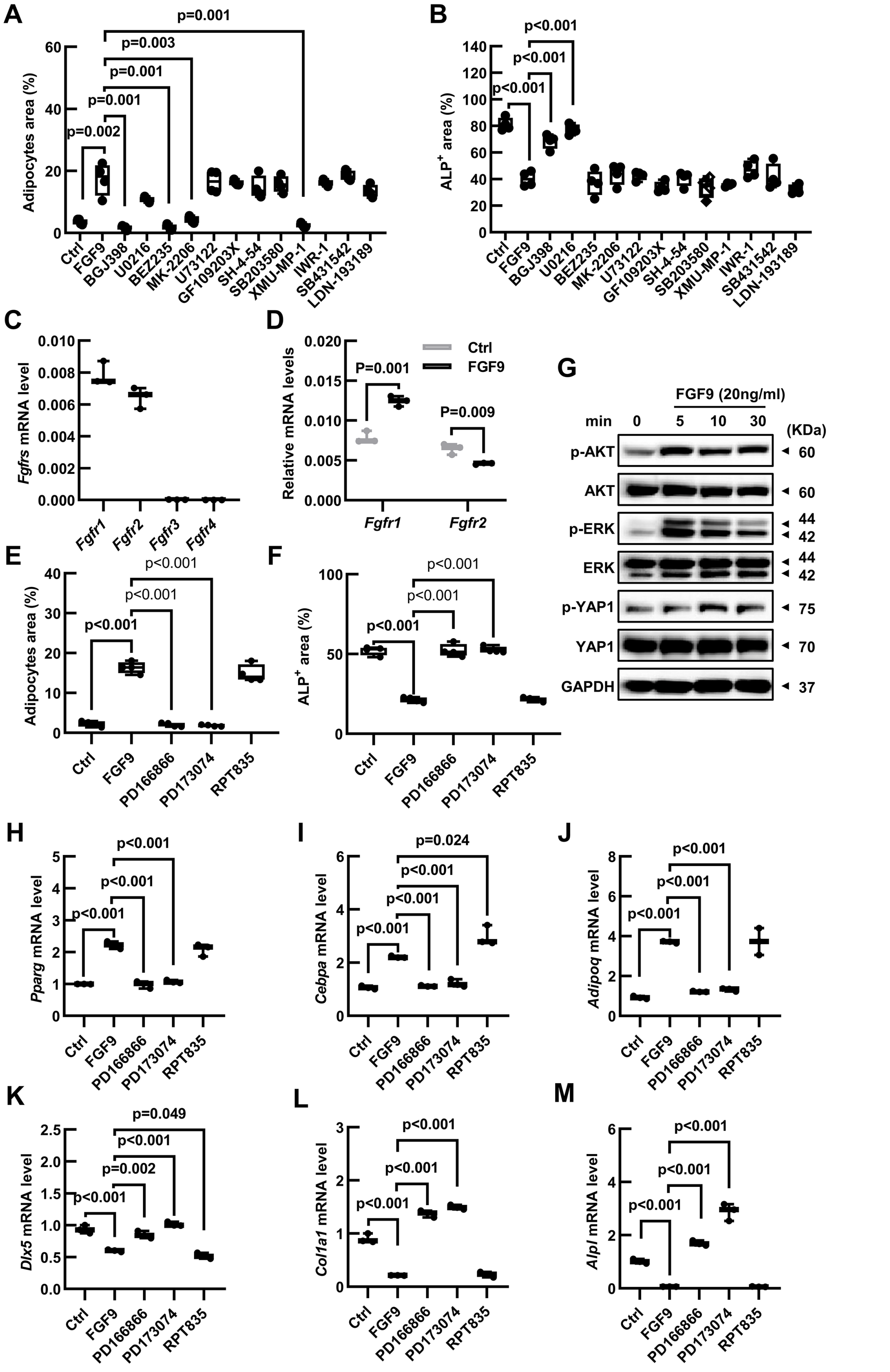

Figure S8. *Fgf9* regulates BMSCs through FGFR1, PI3K/AKT and Hippo pathways. (A-B) Quantification measurement of adipocytes area under AI conditions

(A) and ALP-positive area under OI conditions (B) in BMSCs stimulated with 20 ng/ml FGF9 and inhibitors. n = 4 independent experiments with biological replicates. **(C)** mRNA level of *Fgfr1-4* in BMSCs under CM conditions. **(D)** mRNA expression level of *Fgfr1* and *Fgfr2* in BMSCs with/without FGF9 stimulation. **(E-F)** Quantification measurement of adipocytes area under AI conditions (E) and ALP-positive area under OI conditions (F) in BMSCs stimulated with 20 ng/ml FGF9 and inhibitors (FGFR1, FGFR2). n = 4 independent experiments with biological replicates. **(G)** Immunoblotting analysis showed the phosphorylated and total protein levels of ERK, AKT, and YAP1 in BMSCs stimulated with 20 ng/ml FGF9 for different time periods (0, 5, 10, and 30 min). **(H-M)** Relative mRNA levels of *Cebpa*, *Pparg*, *Adipoq*, *Dlx5*, *Alpl*, and *Col1a1* in BMSCs stimulated with 20 ng/ml FGF9 and inhibitors (FGFR1, FGFR2) under CM conditions for 4 days, n = 3 biological replicates over three independent experiments. Data are analyzed by Student's t-test and shown as boxplots (median ± interquartile range).

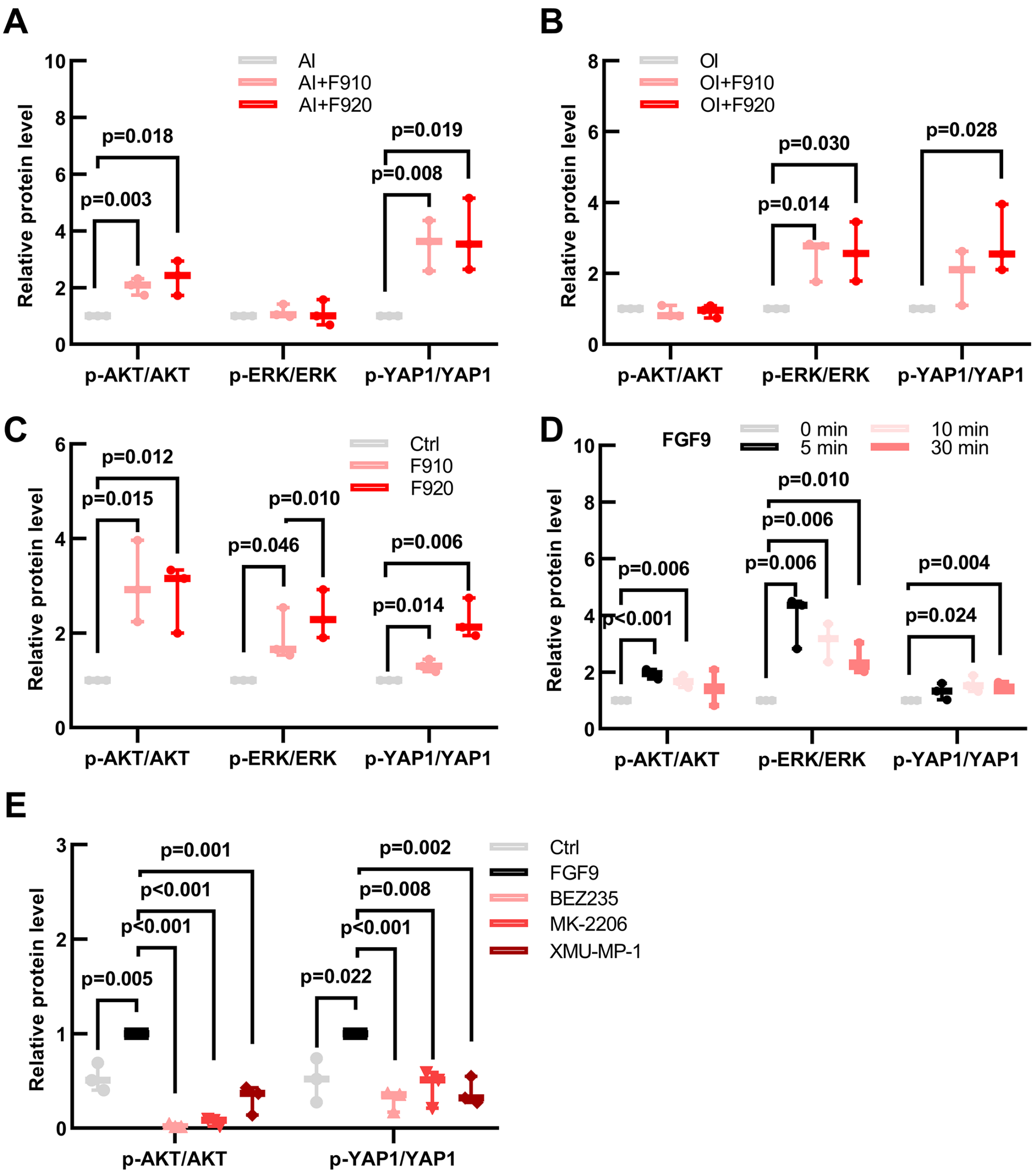

Figure S9. Quantitative results of phosphorylated protein levels from Figure 8. (A-B) Quantification measurement of the phosphorylated protein levels/total protein levels of ERK, AKT, and YAP1 in BMSCs with FGF9 stimulation (0, 10, and 20 ng/ml) under AI (A) or OI (B) conditions for 6 days, referring to Figure 8E. **(C)** Quantification measurement of the phosphorylated/total protein levels of ERK, AKT, and YAP1 in BMSCs stimulated with FGF9 (0, 10, and 20 ng/ml). BMSCs were cultured for 4 days under CM conditions, referring to Figure 8F. **(D)** Quantitative analysis of the phosphorylated protein levels/total protein levels of ERK, AKT, and YAP1 in BMSCs stimulated with 20 ng/ml FGF9 for different time periods (0, 5, 10, and 30 min),

referring to Figure S8G. **(E)** BMSCs were pre-treated with inhibitors for 10 hours and stimulated with 20 ng/ml FGF9 for 10min, and the phosphorylated/total protein levels of AKT and YAP1 in BMSCs were quantified, referring to Figure 8G. n = 3 biological replicates over three independent experiments. Data are analyzed by Student's t-test and shown as boxplots (median ± interquartile range).

Table S1. Inhibitors used for signaling transduction.

	Inhibitor	Targets	Working Con (μM)
1	BGJ398	FGFRs	1.5
2	U0126	MEK	10
3	SB203580	P38	10
4	BEZ235	PI3K, mTOR	1
5	MK-2206	Akt1/2/3	2
6	U73122	PLC-γ	8
7	GF10920X	PKC	1
8	IWR-1-endo	Wnt	10
9	XMU-MP-1	Hippo	5
10	LDN-193189	BMP	0.2
11	SB431542	TGF-β	10
12	SH-4-54	STAT3&5	1
13	PD166866	FGFR1	0.25
14	PD173074	FGFR1	0.1
15	RPT835	FGFR2	10

Table S2. Primers used for mRNA expression detection and genotyping.

Gene	Forward primer	Reverse primer
Osterix	TGATGGGCTGCAAGGGACACTG	TTGGGCTTATAGACATCTTGGGGTAGGA
Runx2	GCGGACGAGGCAAGAGTTTC	AGCGGCGTGGTGGAGTGGAT
Dlx5	CGGCTACTGCTCTCCTACC	ATTCACCATCCTCACCTCTGG
Alpl	ACCAATGTAGCCAAGAATGTCA	CGTTATCCGAGTACCAGTCCC
Col1a1	GCATGAGCCGAAGCTAACCC	GTGGCAGATACAGATCAAGCATACC
Cebpa	GTTAGCCATGTGGTAGGAGACA	CCCAGCCGTTAGTGAAGAGT
Pparg	GAGCACTTCACAAGAAATTACC	AATGCTGGAGAAATCAACTG
Adipoq	CTCCACCCAAGGGAACTTGT	TAGGACCAAGAAGACCTGCATC
Leptin	GTGGCTTTGGTCCTATCTGTC	CGTGTGTGAAATGTCATTGATCC
Fgf9	GCAGTCACGGACTTGGATCAT	TTCTCGTTCATGCCGAGGTAG
Fgfr1	GGATTCTGTGGTGCCTTCTGAC	CAAGTTGTCTGGCCCGAT
Fgfr2	ACCACACCTACCACCTCGAT	GGCATCGCTGTAAACCTTGC
Fgfr3	CTCAGAGGCTGCAAGTGCTAA	CGGGCGAGTCCAATAAGG
Fgfr4	CCGTGGCTGTGAAGATGCTGAA	GCAGGTTGATGATGTTCTTGTGTCTT
β-actin	CTGGCCGGGACCTGACAGACTACC	ATCGGAACCGCTCGTTGCCAATAG
Genotyping	CACTGGGCTCTAACTCTTCTGTCTGC	CTACATCACCATAAGGACCTACCAAGC<br>TCGCATTGTCTGAGTAGGTGTCATTC

Table S3. Antibodies information.

	Name	Cat.NO.	Dilution
1	Anti-p-p44/42 MAPK (Erk1/2) (Thr202/Tyr204) (D13.14.4E)	CST, 4370	WB 1:1000
2	Anti-p44/42 MAPK (Erk1/2) (137F5)	CST, 4695	WB 1:1000
3	Anti-p-Akt (Ser473) (D9E)	CST, 4060	WB 1:1000
4	Anti-AKT	CST, 9272	WB 1:1000
5	Anti-p-YAP1 (Ser397)	Proteintech, 29018-1-AP	WB 1:1000
6	Anti-YAP1	Proteintech, 13584-1-AP	WB 1:1000
7	Anti-C/EBPα	Abcam, ab40764	WB 1:1000
8	Anti-ADIPOQ	Abcam, ab22554	WB 1:1000
9	Anti-PLIN1	Sigma, P1998	WB 1:1000, IF 1:100
10	Anti-PPARγ	CST, 2443	WB 1:1000
11	Anti-RUNX2	Abcam, ab76956	WB 1:1000
12	Anti-ALP	Proteintech, 11187-1-AP	WB 1:1000
13	Anti-OPN	R&D, AF808	WB 1:1000, IF 1:100
14	Anti-COL1	Abcam, ab308221	WB 1:1000
15	Anti-FGF9 (FG9-77)	Invitrogen, MA1-24684	WB 1:1000
16	Anti-S100A4	Proteintech, 16105-1-AP	IF 1:100
17	Anti-GAPDH	Sangon, D110016	WB 1:1000
18	Anti-Goat IgG H&L	Abcam, ab150129	IF 1:500
19	Anti-Rabbit IgG H&L	Invitrogen, A32740	IF 1:500

1
